# The effects of hydrotherapy on athletic ability in children with cerebral palsy: A systematic review and meta-analysis

**DOI:** 10.1371/journal.pone.0325517

**Published:** 2025-06-10

**Authors:** Ye Tao, Ziyi Cao, Min-Chul Shin, Meijia Chen, Shuaiju Han

**Affiliations:** 1 Department of Physical Education, Sejong University, Seoul, South Korea; 2 Department of Physical Education The graduate School of Sangmyung University, Seoul, South Korea; National Institute of Laser Enhanced Sciences (NILES), Cairo University, EGYPT

## Abstract

**Background:**

Cerebral palsy (CP) is a disability caused by brain malformations or injuries occurring from conception to infancy. Hydrotherapy is a popular treatment for children with cerebral palsy and other neuromotor disorders. Despite evidence supporting the efficacy of hydrotherapy for treating children with cerebral palsy, there remains controversy regarding its effectiveness over different follow-up periods and in comparison with other physical therapy methods.

**Objective:**

To compare the effects of hydrotherapy on gross motor abilities, fine motor functions, balance, and muscle tone in children and adolescents with cerebral palsy, and to assess evaluate efficacy across different age stages and treatment durations.

**Methods:**

This meta-analysis was registered with PROSPERO (registration number CRD42024535838). Literature searches were conducted in databases including CNKI, VIP, WanFang, Web of Science, PubMed, Embase, and the Cochrane Library starting from June 2024. Randomized controlled trials (RCTs) that assessed the effects of hydrotherapy on gross motor functions, fine motor functions, balance, and muscle tone in children and adolescents with cerebral palsy were included. RCTs were evaluated for quality using the Cochrane risk of bias tool. Outcomes were analyzed by calculating mean difference (MD) or standardized mean difference (SMD) along with their 95% confidence intervals (CIs).

**Results:**

Sixteen studies were included, assessing the impact of hydrotherapy compared to conventional care on gross motor functions in children and adolescents with cerebral palsy. The findings indicated that hydrotherapy significantly improved gross motor functions [SMD = 0.41, 95% CI = 0.15–0.68, I^2^ = 59.5%, p < 0.05], with consistent effects observed in children aged ≤6 years [SMD = 0.42, 95% CI = 0.16–0.68, I^2^ = 38.2%, p < 0.05] and those aged >6 years [SMD = 0.43, 95% CI = 0.22–0.63, I^2^ = 59.5%, p < 0.05]. Subgroup analysis based on intervention duration revealed that programs lasting more than 10 weeks were associated with significant improvements [SMD = 0.48, 95% CI = 0.31–0.66, I^2^ = 65.1%, p < 0.05], whereas no significant effects were found in interventions lasting 10 weeks or less [SMD = 0.14, 95% CI = –0.26–0.53, I^2^ = 35.6%, p > 0.05]. Hydrotherapy demonstrated a certain positive effect on fine motor functions [SMD = 0.78, 95% CI = 0.46–1.10, I^2^ = 46.4%, p > 0.05].In contrast, no statistically significant improvements were observed in balance [SMD = 0.64, 95% CI = –0.05–1.34, I^2^ = 80.7%, p > 0.05] or muscle tone [SMD = –0.45, 95% CI = –0.98–0.07, I^2^ = 58.2%, p > 0.05].

**Conclusion:**

The results indicate that hydrotherapy is more effective than conventional treatment in improving gross motor functions in children and adolescents with cerebral palsy, with consistent benefits observed across different age groups and in interventions of longer duration. Hydrotherapy also showed a positive trend in enhancing fine motor functions, although no significant improvements were observed in balance or muscle tone.

## Introduction

Cerebral palsy (CP) is a disability caused by abnormal development or damage to the brain during prenatal development or infancy [1,2]. The World Health Organization reports that the incidence of cerebral palsy in developed countries ranges from 0.2% to 0.3%, establishing it as a prevalent condition worldwide with an estimated 2–3 cases per 1,000 individuals [3]. This prevalence marks cerebral palsy as a significant global public health issue [4]. Children with cerebral palsy often face cognitive and communication challenges that limit their participation in family, educational, and social activities. Additionally, central nervous system injuries in these children may result in muscle spasms, atrophy, and weakness, which impede motor skill development [5,6]. Typically, the motor functions of children with cerebral palsy are inferior to those of their peers. Prolonged inactivity in sports and physical activities can lead to severe physical and mental health outcomes for these children [7]. Thus, enhancing motor function in children with cerebral palsy is of paramount importance.

A variety of therapeutic interventions are currently used in the rehabilitation training of children and adolescents with cerebral palsy. These include strengthening exercises, stretching, balance training, treadmill training, and functional task-oriented training [8]. Research shows that physical and occupational therapy can improve muscle strength and endurance, enhance joint mobility, and facilitate activities of daily living and functional performance [9]. The primary focus of rehabilitation for children and adolescents with cerebral palsy is to improve health, functional activities, and participation [10].

Hydrotherapy is a widely recognized alternative treatment for children with neuromotor disorders, such as cerebral palsy. It facilitates exercise, assisting in managing these conditions. Hydrotherapy enhances children’s health, functional activities, and overall participation [11]. The buoyancy and reduced gravity of water alleviate joint stress, making it a favored complementary therapy for individuals with cerebral palsy. Research indicates that hydrotherapy addresses various issues, including fatigue, obesity, stress, and anxiety, while also enhancing immune function, alleviating pain, and restoring cardiopulmonary capacity [7]. Additionally, it reduces muscle spasm, improves tolerance to multisensory stimuli, and enhances blood circulation in children with cerebral palsy [12]. Many studies have shown that hydrotherapy improves gross motor functions, fine motor functions, balance, and muscle tone [13], offering unique advantages in the rehabilitation of children with cerebral palsy.

Current research primarily focuses on the health impacts of hydrotherapy on individuals with cerebral palsy or its effects on their quality of life. For instance, Masoud Khalaji et al. [14] found that when hydrotherapy was combined with conventional rehabilitation methods, it had a positive impact on the quality of life of individuals with cerebral palsy. Although several studies have explored the effects of hydrotherapy on patients with cerebral palsy, research specifically analyzing its impact on motor function in children remains limited. Catalina Tapia et al. found that hydrotherapy significantly improved gross motor function and aquatic functions in children with cerebral palsy, while also enhancing their social interaction functions [15]. Similarly, Tina Makris et al. reported significant improvements in physical quality of life among children with cerebral palsy [16]. Elton Pauluka et al. demonstrated that aquatic interventions positively influence gross motor function in children and adolescents with cerebral palsy, although the studies lacked specific guidance on optimal intervention dosage and intensity [17]. Silvia Faccioli et al. confirmed the effectiveness of aquatic exercise in improving gross motor functions and emphasized the need for future research to explore effects across different age groups and GMFCS levels [18]. Likewise, Meysam Roostaei et al. concluded that aquatic interventions are a safe and effective rehabilitation approach for children with cerebral palsy [19]; however, further studies are needed to determine the most appropriate intervention strategies and dosage parameters.

Through this systematic review and meta-analysis, we aim to evaluate the effectiveness of hydrotherapy in enhancing the motor capabilities of children with cerebral palsy. We hypothesize that hydrotherapy can effectively improve the motor functions of children with cerebral palsy.

## Methods

In conducting this systematic review, the researchers followed the Preferred Reporting Items for Systematic Reviews and Meta-Analyses (PRISMA) standards and registered the protocol on the international prospective systematic review registration platform PROSPERO (Record ID: CRD42024535838).

### Search strategy

This study commenced in June 2024 and involved a comprehensive search of seven databases for search of databases. These databases included CNKI, VIP, WanFang Database, Web of Science, PubMed, Embase, and Cochrane Library. The search terms were tailored to the specific characteristics of each database and were executed by two researchers. Keywords or MeSH terms used included: cerebral palsy or CP, mixed-type cerebral palsy, hemiplegic Cerebral palsy, infantile or Rolandic Cerebral palsy, pediatric diseases, spastic cerebral palsy, hydrotherapy, massage bath, Watsu therapy, Halliwick, aquatic exercise, exercise, motor ability, physical ability, and physical training. Details of the search strategies for each database are provided in S1 File. An initial literature search was conducted by a team member (YT), who cross-verified the findings with other authors. Following this, the titles and abstracts of identified articles were imported into EndNote for full-text screening.

### Inclusion criteria and research selection

The inclusion criteria for this systematic review and meta-analysis were developed based on the PICOS framework, taking into account current gaps in literature. The objective of this study is to evaluate the effectiveness of hydrotherapy in improving motor functions in children and adolescents with cerebral palsy. The specific research question is defined as follows:

- Population (P): Children and adolescents (0–23 years) diagnosed with cerebral palsy, regardless of gender or CP subtype. Studies that included participants with mixed neurological disorders without separate analysis for CP were excluded.
- Intervention (I): Hydrotherapy, defined as structured, evidence-based physical activities or exercises performed in water, including aquatic walking, swimming training, balance exercises, and resistance training. Studies lacking sufficient detail about the intervention or involving a single session of aquatic exercise were excluded.
- Comparator (C): Conventional rehabilitation methods (land-based therapies) or waiting list controls. Conventional therapies may include strength training, stretching, balance exercises, and motor coordination training. Studies using only recreational or leisure-based activities as control interventions were excluded.
- Outcome (O): Motor ability outcomes, including gross motor functions, fine motor functions, balance, and muscle tone, with sufficient quantitative data provided (e.g., means and standard deviations) to allow for the calculation of effect sizes such as Hedges’ g [20]
- Study design: Only randomized controlled trials (RCTs) directly comparing hydrotherapy with conventional treatments or a waiting list were included [21].

Exclusion Criteria:

(a)  Duplicate publications.
(b)  Studies in which hydrotherapy was not the primary intervention or was combined with other therapies.
(c)  Animal studies.
(d)  Studies with unclear methodological design or insufficient rigor.
(e)  Reviews, case-control studies, case reports, expert opinions, and descriptive studies.

### Study selection

Screening was conducted by two researchers using predefined inclusion and exclusion criteria. In cases where consensus was not reached, discrepancies were resolved through discussion. If disagreements persisted, a third researcher made the final decision. References for the included studies were checked to identify additional potentially relevant studies.

### Data extraction

Data extraction was performed using a customized Excel (Microsoft Inc.) data extraction tool. The first and second authors jointly extracted data [22]. Extracted information included: author details (first author, country, year of publication); characteristics of the study population (gender, age, type of condition, GMFCS level) Gross Motor Function Classification System (GMFCS); experimental methods (equipment, interventions, duration of treatment); and outcome measurement methods and indicators. Only quantitative motor function outcomes were examined in accordance with the study objectives. The outcome indicators included gross motor functions measured by the Gross Motor Function Measure (GMFM), fine motor functions assessed by the (FM-FM), balance assessed by the Pediatric Berg’s Balance Scale (PBS), and muscle tone assessed by the Modified Ashworth Scale (MAS).

### Quality assessment

The quality of the studies included in the analysis was assessed in two stages:

Methodological quality was assessed using the Cochrane risk of bias tool. Two independent researchers (YT and SJH) evaluated the studies, focusing on the following aspects: (1) randomization process, (2) deviations from intended interventions, (3) incomplete outcome data, (4) outcome measurement, and (5) selection of reported outcomes. In cases of inconsistency, a third researcher (YZC) was consulted to reach a consensus. The studies were categorized as “low risk of bias,” “unclear risk of bias,” or “high risk of bias.”

### Quality of evidence

The overall quality of evidence was assessed using the Grading of Recommendations Assessment, Development, and Evaluation (GRADE) system. This system evaluates the certainty of the evidence based on risk of bias, inconsistency, indirectness, imprecision, and other considerations. The result was categorized as high, moderate, low, and very low certainty of evidence.

### Statistical analysis

The meta-analysis was conducted using Stata-MP 14 and Revman 5.3 software. For continuous variables, Standardized means difference (SMD). If studies provided medians and ranges instead of standard deviations (SD), methods developed by Luo et al. and Wan et al. were used to calculate the SD [23,24]. If heterogeneity among the studies was not significant (P > 0.1, I^2^ ≤ 50%), a fixed-effect model was applied; if significant (P ≤ 0.1, I^2^ > 50%) [25], a random-effects model was utilized. According to Cochrane Collaboration guidelines, heterogeneity is interpreted with I^2^ values of 25%, 50%, and 75% indicating low, moderate, and high heterogeneity, respectively. When I^2^ ≥ 50%, sensitivity analysis was conducted to test the stability of the results, and subgroup analyses were performed based on the characteristics of the literature. Additionally, Begg’s and Egger’s tests were used, along with funnel plots, to quantitatively analyze publication bias in the included studies. Results were presented in a forest plot.

## Results

### Study selection

**Fig 1:**
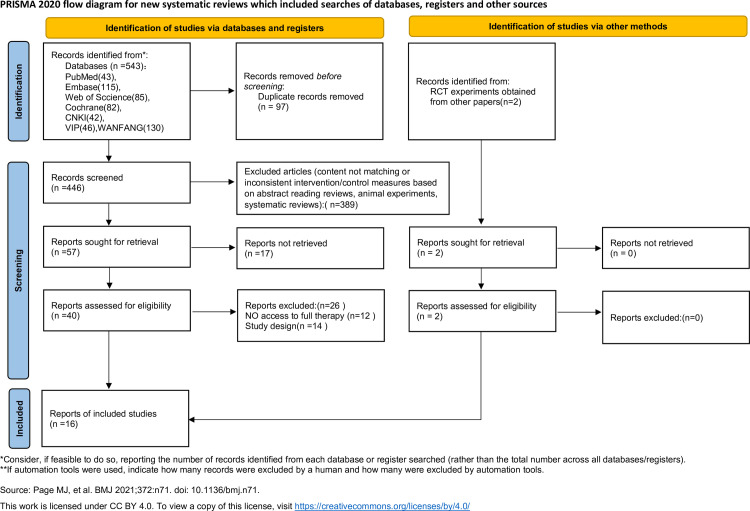
PRISMA flow diagram of the study selection.

In this analysis, 545 relevant articles were identified through searches of seven databases, and an additional 2 articles were obtained from external sources. After removing duplicates, 446 articles were retained. Subsequently, articles that did not match the topic or had inconsistent intervention or control measures, including review articles, animal studies, and systematic reviews were excluded based on abstract screening, leaving 57 articles. Further screening excluded 17 articles due to the inability to retrieve full texts, leaving 40 articles. Ultimately, studies lacking complete treatment data or containing methodological limitations were excluded, resulting in 16 RCTs being included in the analysis [26–41]. The study selection process is visually presented in the PRISMA diagram (Fig 1).

### General characteristics and quality assessment of included studies

The included studies were RCTs conducted between 2009 and 2023, involving a total of 634 participants, with ages ranging from 1 to 23 years. Among these, 319 participants were in the hydrotherapy group, and 315 were in the conventional training group. The gender distribution consisted of 319 males and 275 females. Not all studies reported the GMFCS levels prior to intervention. In the hydrotherapy group, the GMFCS levels were distributed as follows: 21 at Level I, 47 at Level II, 27 at Level III, 23 at Level IV, and 8 at Level V. Correspondingly, in the conventional training group, there were 19 at Level I, 32 at Level II, 37 at Level III, 22 at Level IV, and 8 at Level V. The minimum sample size in these studies was 6, and the maximum sample size was 55. The studies primarily assessed the efficacy of aquatic therapy alone compared to conventional training, as well as aquatic therapy combined with conventional training versus conventional training alone (Table 1). The studies were conducted in various regions, including Turkey, Nigeria, Egypt, Greece, the UK, Serbia, South Korea, and China.

**Table 1:** the characteristics of the included studies in the meta-analysis.

Study	Country	Participant Information [Number; Gender; Age (years old)]	GMFCS Level (Number)	experimental design Intervention Group	experimental design Control Group	Primary Outcome Measures
Adar, 2017 [26]	Turkey	Hydrotherapy group: 17; 8 male, 9 female; 10.1 ± 2.4 Conventional training group: 15; 6male, 9 female; 9.3 ± 1.9	Hydrotherapy group: 6 in Level I, 6 in Level II, 3 in Level III and 2 in Level IV Conventional training group:6 in Level I, 2 in Level II, 4 in Level III and 3 in Level IV	Aquatic exercise(60 minutes per day, 5 times a week, for 6 weeks, a total of 30 sessions)	Land-based exercise(60 minutes per day, 5 times a week, for 6 weeks, a total of 30 sessions)	MAS; TUG; GMFM-88;WeeFIM; Ultrasonographic assessment of spastic gastrocnemius muscle
Akinola, 2019 [27]	Nigeria	Hydrotherapy group: 15; 4.93 ± 1.98 Conventional training group: 15; 5.41 ± 2.85	Hydrotherapy group: 1 in Level II, 5 in Level III an, 7 in Level IV and 2 in Level V Conventional training group:6 in Level III and 9 in Level IV	Aquatic exercise(100 minutes per day, 2 times a week, for 10 weeks, a total of 20sessions)	Land-based exercise(100 minutes per day, 2 times a week, for 10 weeks, a total of 20sessions)	GMFM-88
Badawy, 2016 [28]	Egypt	Hydrotherapy group: 15; 10 male, 5 female; 7.8 ± 1.01 Conventional training group: 15; 9male, 6 female; 7.73 ± 1.16	Hydrotherapy group: 7 in Level II and 8 in Level III Conventional training group:6 in Level II and 9 in Level III	Aquatic exercise(60 minutes per day, 3 times a week, for 12 weeks)	Land-based exercise(60minutes per day,3 times a week, for 12 weeks)	Balance;Kinematic gait
Chrysagis, 2009 [29]	Greece	Hydrotherapy group: 6; 4 male, 2 female; 16.0 ± 2.9; Conventional training group: 6; 3male, 3 female; 16.66 ± 2.65		Swimming program+Physiotherapy(60minutes per day,2 times a week, for 10 weeks)	Physiotherapy(60minutes per day,2 times a week, for 10 weeks)	GMFM-88;MAS;ROM
Declerck, 2016 [30]	England	Hydrotherapy group group:7; 5 male,2 female;7–17 Conventional training group: 7; 3male,4 female; 7–17	Hydrotherapy group group:1 in Level I and 6 in Level II Conventional training group:2 in Level I, 4 in Level II and 1 in Level III	Swimming program+Physiotherapy(40–50 minutes per day,2 times a week, for 10 weeks)	Physiotherapy(40–50 minutes per day,2 times a week, for 10 weeks)	WOTA
Dimitrijevic, 2012 [31]	Serbia	Hydrotherapy group group:14; 10 male, 4 female;9.21 ± 2.45 Conventional training group: 13; 7male,6female;9.92 ± 2.32	Hydrotherapy group group:6 in Level I, 3 in Level II, 2 in Level III,1 in Level IV and in 2 Level V Conventional training group:4 in Level I, 3 in Level II, 2 in Level III,1 in Level IV and in 3 Level V	aquatic skills(55minutes per day,2 times a week, for 6 weeks)	dry-land motor skills(55 minutes per day,2 times a week, for 6 weeks)	GMFM-88;WOTA
Hamed, 2023 [32]	Egypt	Hydrotherapy group group: 17; 12 male, 5 female; 4.62 ± 0.41 Conventional training group: 17; 10male, 7 female;4.51 ± 0.4	n ≤ 2 in Level III	Halliwick-hydrotherapy(45 minutes per day, 3 times a week, for 12)	Physiotherapy(45 minutes per day, 3times a week, for 12 weeks)	GMFM-88
Kang, 2012 [33]	South Korea	Hydrotherapy group: 15;11 male,4 female; 8.40 ± 2.19 Conventional training group: 15; 8 male, 7 female; 8.06 ± 2.4	Hydrotherapy group: 9 in Level II and 6 in Level IV Conventional training group: 10 in Level II Iand 5 in Level IV	Halliwick-hydrotherapy(30 minutes per day, 3 times a week, for 8)	Land-based exercise(30 minutes per day, 3times a week, for 8 weeks)	ROM; MAS; PBS; GMFM-88
Chandolias. K, 2022 [34]	Greece	Hydrotherapy group: 28; 14 male, 14 female; The average age was 7.53 years Conventional training group: 26; 12male, 14 female; The average age was 7.53 years	Hydrotherapy group:4 in Level I, 8 in Level II, 5 in Level III, 7 in Level IV and in 4 Level V Conventional training group:4 in Level I,9 in Level II, 8 in Level III, 3 in Level IV and in 2 Level V	Halliwick-hydrotherapy+Physiotherapy(2 times a week, for 3 months)	Physiotherapy(40–50 minutes per day,2 times a week, for 3 months)	GMFM-88
Luo, 2018 [35]	China	Hydrotherapy group: 43; 23 male, 20 female; 22.4 ± 8.7 Conventional training group: 43; 24male, 19 female;22.9 ± 8.5		Conventional Bobath Therapy + Hydrotherapy(60minutes per day 5 times a week, for 3 months)	Conventional Bobath Therapy(60minutes per day 5 times a week, for 3 months)	GMFM-88;FM‐FM;ntegrated Electromyography (iEMG)
Olama, 2015 [36]	Egypt	Hydrotherapy group: 15; 6 male, 9 female; 5.81 ± 1.79 Conventional training group: 15; 7male, 8female;5.76 ± 1.41		exercise therapy Program+ aquatic exercise program(45minutes per day,2 times a week, for 20 weeks)	exercise therapy Program(45minutes per day,2 times a week, for 20 weeks)	Hoffman reflex and Myogenic response (H/M) ratio;degree of ankle excursion
Song, 2015 [37]	China	Hydrotherapy group: 30;17 male, 13 female; 5.81 ± 1.79 Conventional training group: 30; 20male, 10 female;5.76 ± 1.41		Conventional Training + Hydrotherapy(70minutes per day 1 times a week, for 3months)	Conventional Training + (40minutes per day 2 times a week, for 3months)	GMFM-88; Quadriceps Thickness; MAS;
Zhao, 2022 [38]	China	Hydrotherapy group: 30;20 male, 10 female; 3.40 ± 0.3 Conventional training group: 30; 17male, 13 female;3.52 ± 0.3		Conventional Therapy + Hydrotherapy(60minutes per day 2 times a week, for 3 months)	Conventional Therapy + Hydrotherapy(60minutes per day 2 times a week, for 3 months)	GMFM-88; 6MWT; BBS; MAS
Zhang, 2022 [39]	China	Hydrotherapy group: 40;23 male, 17 female; 3.25 ± 0.56 Conventional training group: 40; 21male, 19 female;3.48 ± 0.2 9		Halliwick-hydrotherapy+Conventional Training(60minutes per day 4times a week, for 3 months)	Conventional Training + (60minutes per day 4 times a week, for 3 months)	GMFM-88; Balance; Lower Limb Joint Range of Motion; FMFM、PDMS-FM
Zhu, 2022 [40]	China	Hydrotherapy group: 55;25 male, 30female; 4.26 ± 0.82Conventional training group:55; 27male, 28 female;4.19 ± 0.33		Conventional Therapy + Hydrotherapy(60minutes per day 2 times a week, for 3 months)	Conventional Therapy + Hydrotherapy(60minutes per day 2 times a week, for 3 months)	GMFM-88; Lower Limb Joint Range of Motion
Zhong, 2023 [41]	China	Hydrotherapy group: 12;6 male, 6female; 4.1 ± 1 Conventional training group:13; 5male, 8 female;3.7 ± 1	Hydrotherapy group:3 in Level I, 7 in Level II and 2 in Level III Conventional training group:3 in Level I, 8 in Level I Iand2 in Level III	Conventional Training + Hydrotherapy(75minutes per day 5 times a week, for 12 weeks	Conventional Training(75minutes per day 5 times a week, for 12 weeks	GMFM-88; Lower Limb Joint Range of Motion; MAS

### Risk of bias of individual studies

**Fig 2:**
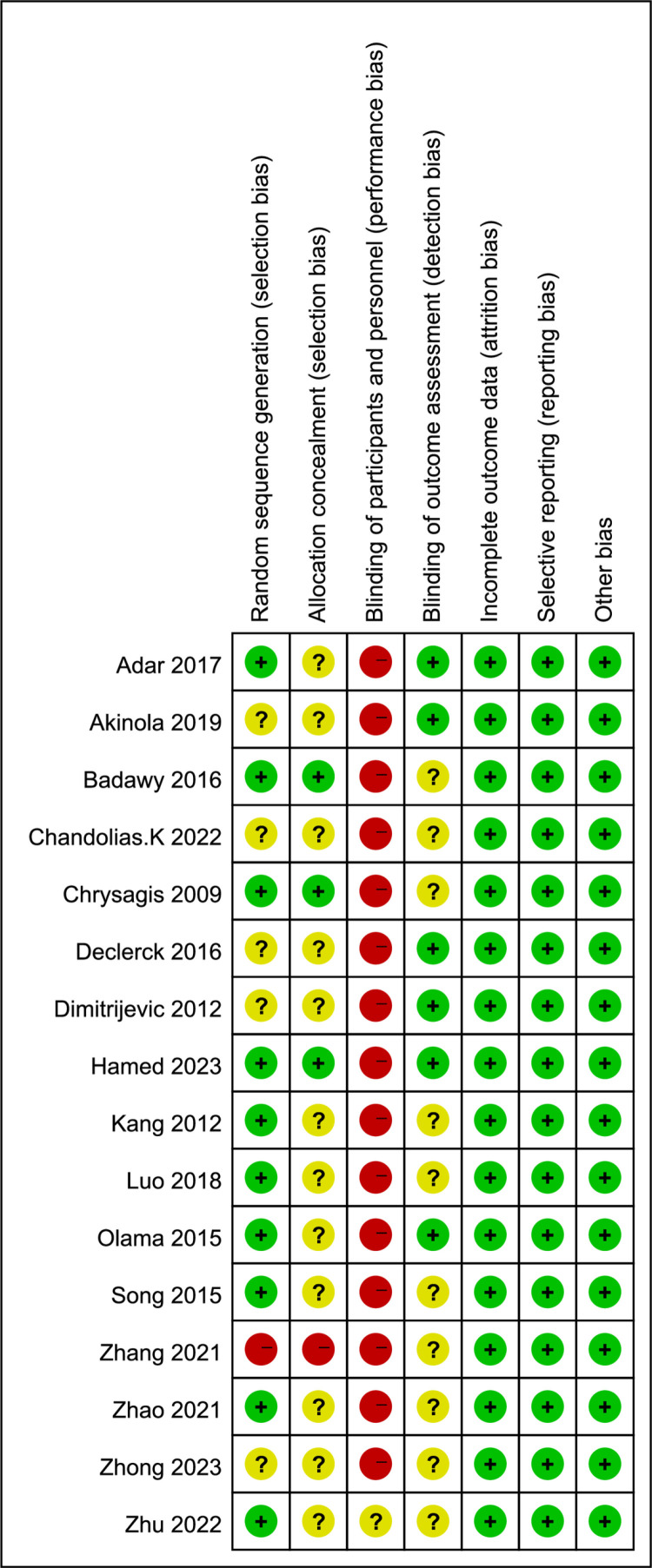
Risk of bias summary.

**Fig 3:**
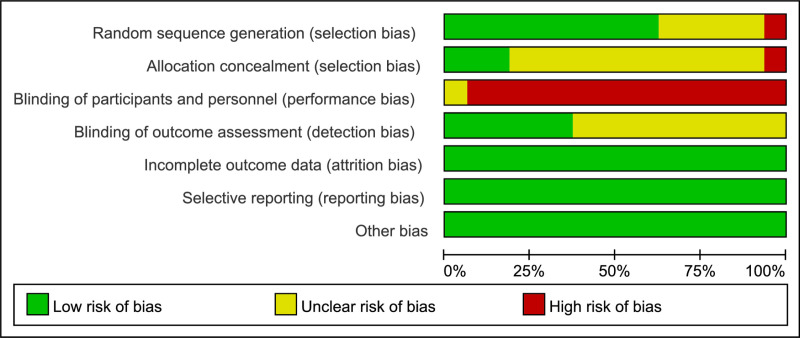
Risk of bias graph.

All 16 reviewed studies included the terms “random” or “randomized controlled trial”. Nine studies outlined unique randomization methods, such as random number tables and computer randomization. Three studies addressed allocation concealment. Due to the nature of the participants, children and adolescents with cerebral palsy, it was impractical to blind participants and staff fully. However, six studies detailed blinding procedures for assessors, demonstrating a low risk of bias across other evaluated outcomes. These findings are illustrated in the Figs 2 and 3.

### Meta-analysis results

#### The effect of hydrotherapy on gross motor functions.

**Fig 4:**
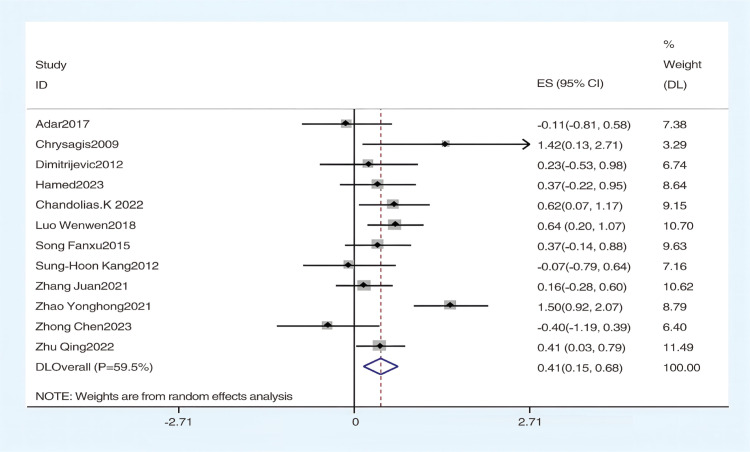
Forest plot of gross motor function.

**Fig 5:**
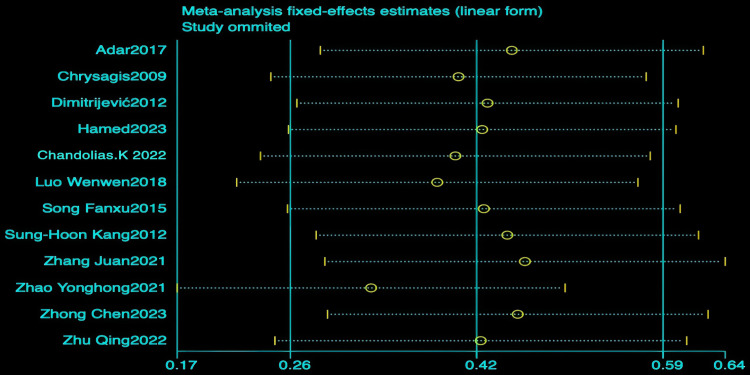
Gross motor function Sensitivity Analysis Chart.

**Fig 6:**
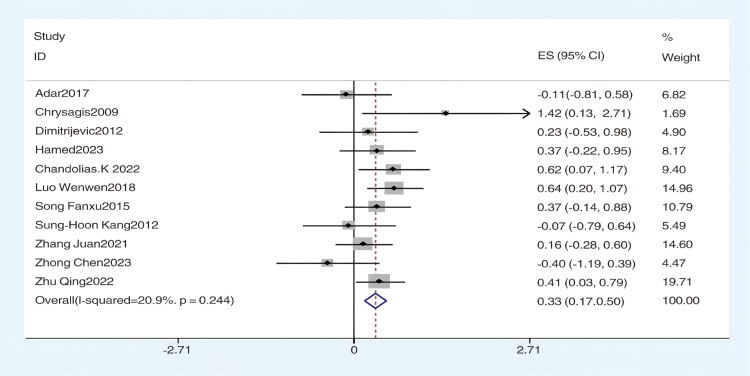
Forest chart of gross motor function after deleting articles with high sensitivity.

**Fig 7:**
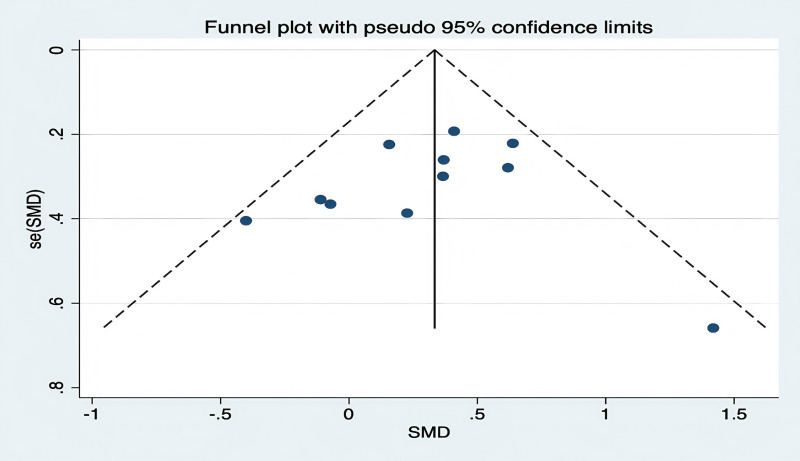
Funnel plot of gross motor function.

**Fig 8:**
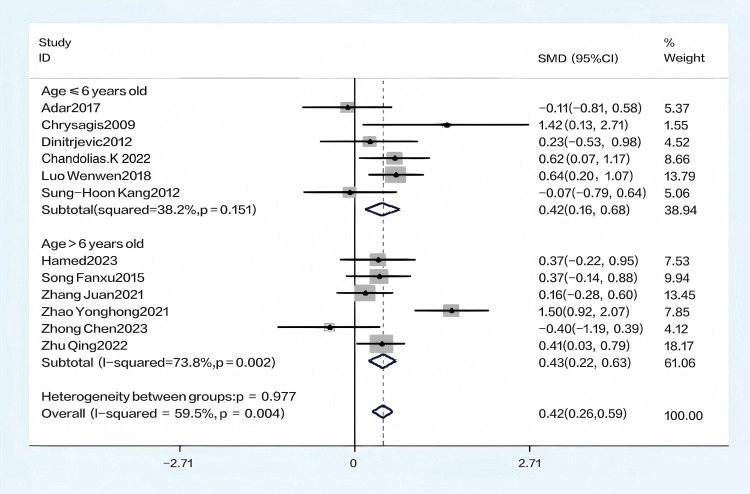
Forest plot of analyses was performed based on age.

**Fig 9:**
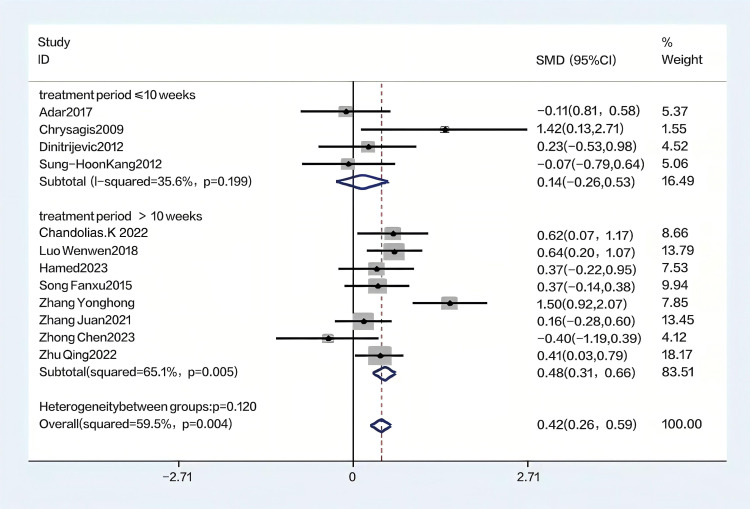
Forest plot of subgroup analysis based on treatment period classification.

Using the GMFM-88 scale to assess the impact of hydrotherapy on gross motor functions in children and adolescents with cerebral palsy, 12 of the 16 included studies [26,29,31–35,37–41] focused on improving gross motor functions in this population. The results showed (SMD = 0.41, 95% CI = 0.15–0.68, I^2^ = 59.5%, p < 0.05) (Fig 4), indicating that the hydrotherapy group significantly outperformed the conventional training group in terms of gross motor function. Variations in participant age or trial duration prompted further subgroup analyses based on these variables. Due to high heterogeneity, a sensitivity analysis was conducted, revealing that the study by Zhao et al. significantly impacted heterogeneity (Fig 5) [26,29,31–35,37,39–41]; excluding this study resulted in (SMD = 0.33, 95% CI = 0.17–0.50, I^2^ = 20.9%, p < 0.05) (Fig 6), where the hydrotherapy group again significantly outperformed the conventional training group in terms of gross motor function. To assess potential publication bias, a funnel plot was generated (Fig 7), which displayed symmetry (p = 0.631), indicating no significant publication bias among the reviewed studies. Subgroup analysis further explored the effects of age and treatment duration. In the age analysis, six studies of children aged 6 and under showed a total effect size for hydrotherapy of (SMD = 0.42, 95% CI = 0.16–0.68, I^2^ = 38.2%, p < 0.05) (Fig 8) [26,29,31,33–35]; studies of children older than 6 years revealed (SMD = 0.43, 95% CI = 0.22–0.63, I^2^ = 59.5%, p < 0.05) [32,34,35,37–41], both indicating a positive impact of hydrotherapy. In the treatment duration analysis, four studies with a duration of 10 weeks or less showed (SMD = 0.14, 95% CI = −0.26–0.53, I^2^ = 35.6%, p > 0.05) [32,37–41]; however, seven trials lasting over 10 weeks demonstrated (SMD = 0.48, 95% CI = 0.31–0.66, I^2^ = 65.1%, p > 0.05) (Fig 9) [32,34,35,37–41]. These results suggest that hydrotherapy has a positive effect on gross motor functions in children with cerebral palsy, though treatment duration may influence outcomes. The certainty of this evidence was judged to be low overall S1 Table.

#### The effect of hydrotherapy on fine motor functions.

**Fig 10:**
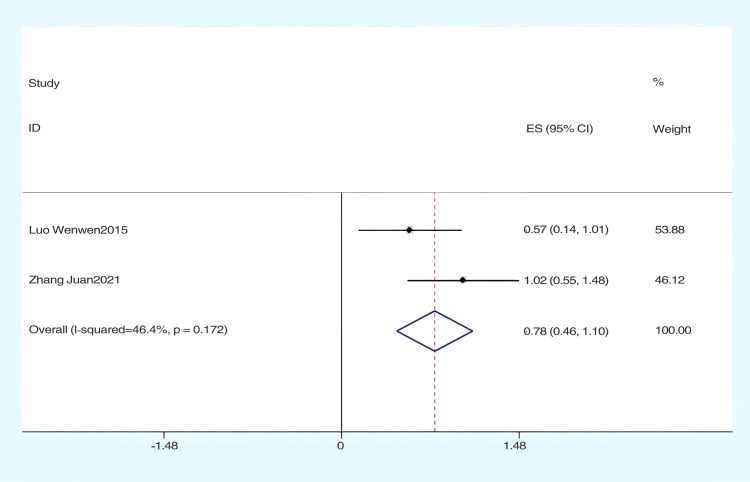
Forest chart of fine motor function.

The analysis of two studies [35,39] assessed the impact of hydrotherapy on fine motor functions in children and adolescents with cerebral palsy. The results indicated (SMD = 0.78, 95% CI = 0.46–1.10, I^2 ^= 46.4%, p > 0.05) (Fig 10), showing that the hydrotherapy group significantly outperformed the conventional training group in terms of fine motor functions. Additionally, bias testing using Egger’s test yielded a p-value of 0.317. The certainty of this evidence was judged to be very low overall S1 Table.

#### The effect of hydrotherapy on balance.

**Fig 11:**
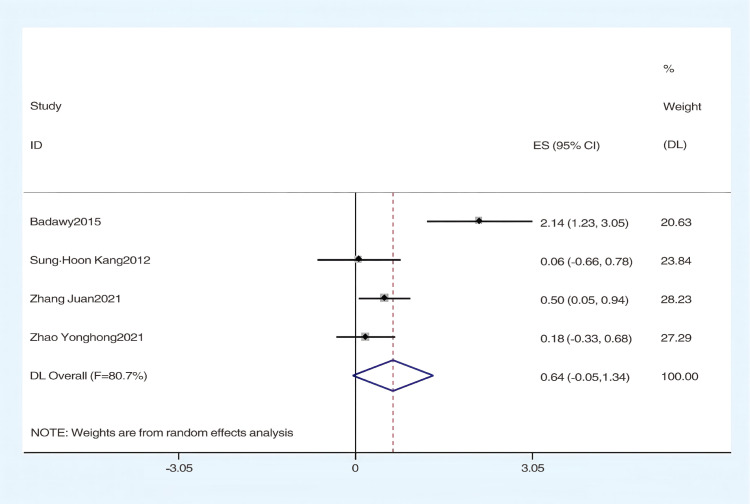
Forest chart of balance.

**Fig 12:**
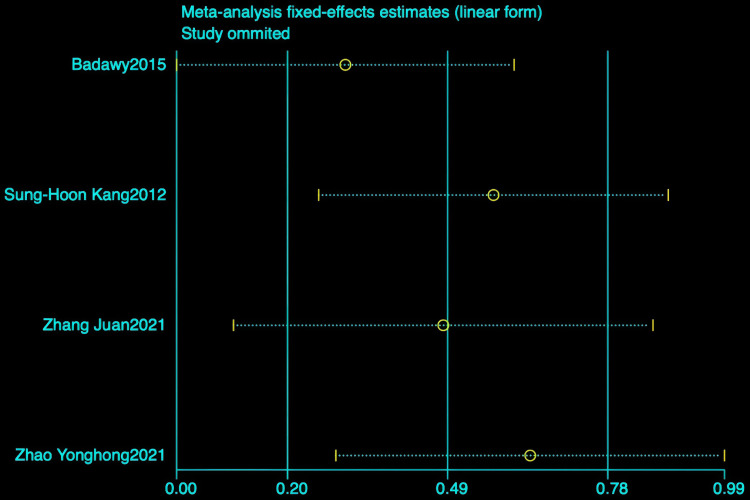
Balance Sensitivity Analysis Chart.

**Fig 13:**
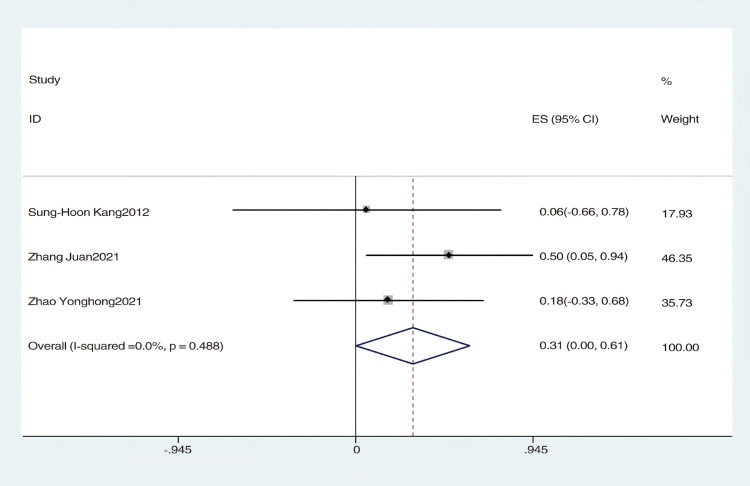
Forest chart of balance after deleting articles with high sensitivity.

An analysis of four studies assessed the impact of hydrotherapy on balance in children and adolescents with cerebral palsy [28,33,38,39]. The results indicated (SMD = 0.64, 95% CI = −0.05–1.34, I^2^ = 80.7%, p > 0.05) (Fig 11), showing that the hydrotherapy group slightly outperformed the conventional training group in terms of balance, though the difference was not statistically significant. High heterogeneity, attributed to variations in participant age or trial design, necessitated a sensitivity analysis. This analysis identified the study by Badawy et al. as significantly impacting heterogeneity (Fig 12); excluding this study resulted in (SMD = 0.31, 95% CI = −0.05–0.67, I^2^ = 50.9%, p > 0.05), and while heterogeneity decreased, the results remained non-significant (Fig 13) [33,38,39]. These findings suggest hydrotherapy may have a positive effect on balance, but further research is needed to validate this due to high heterogeneity. The certainty of this evidence was judged to be very low overall S1 Table.

#### The effect of hydrotherapy on muscle tone.

**Fig 14:**
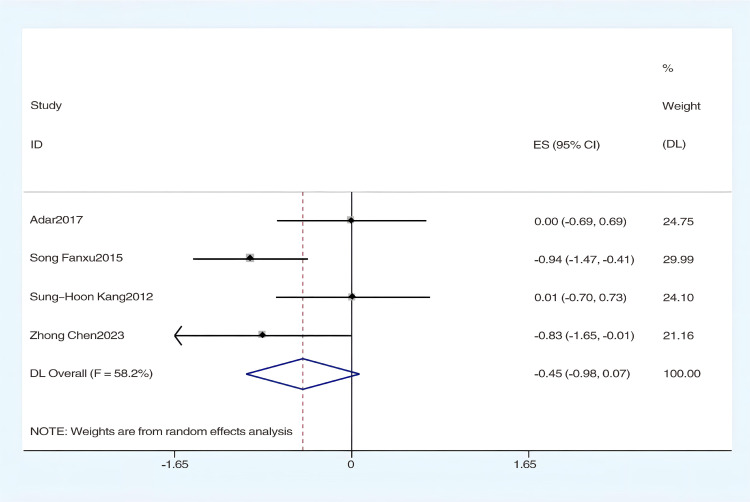
Forest chart of muscle tone.

**Fig 15:**
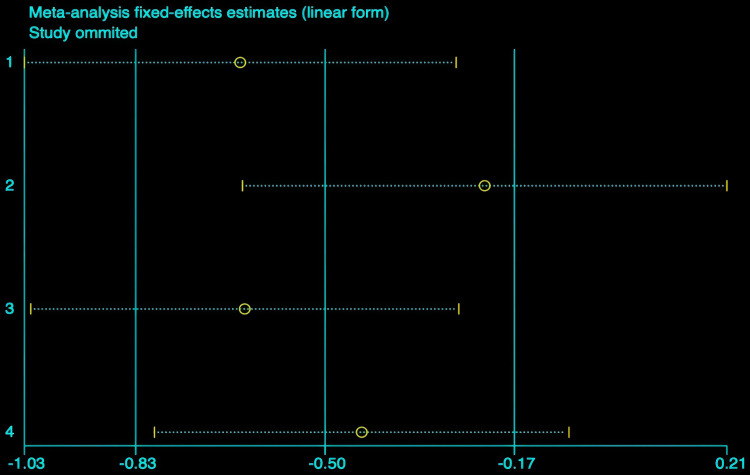
Muscle tone Sensitivity Analysis Chart.

**Fig 16:**
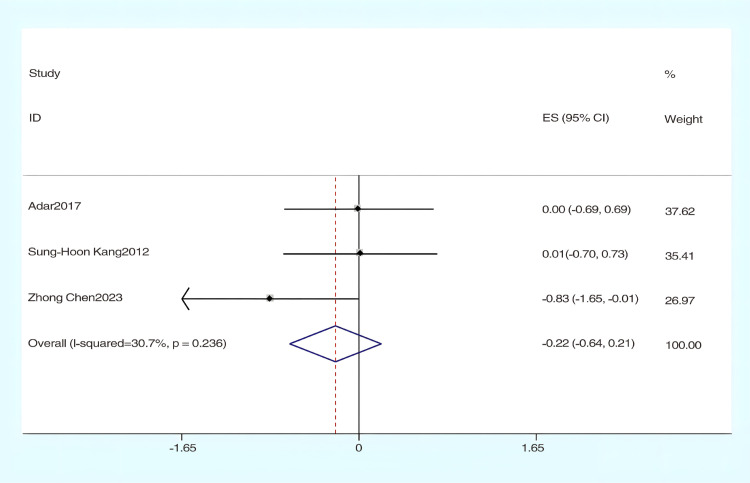
Forest chart of muscle tone after deleting articles with high sensitivity.

An analysis of four studies evaluated the impact of hydrotherapy on muscle tone in children and adolescents with cerebral palsy [26,33,37,40]. The results indicated (SMD = −0.45, 95% CI = −0.98–0.07, I^2^ = 58.2%, p > 0.05) (Fig 14), showing that the hydrotherapy group experienced an improvement in muscle tone compared to the conventional training group, though the difference was not statistically significant. Sensitivity analysis revealed that the study by Song et al. significantly influenced heterogeneity (Fig 15); excluding this study resulted in (SMD = −0.22, 95% CI = −0.64–0.21, I^2^ = 50.9%, p > 0.05) (Fig 16) [26,33,41]. The certainty of this evidence was judged to be very low overall S1 Table.

## Discussion

This review systematically assessed and conducted a meta-analysis on the effects of hydrotherapy on motor functions in children and adolescents with cerebral Palsy. The findings suggest that hydrotherapy positively impacts gross motor functions, fine motor functions, balance, and muscle tone.

In terms of gross motor functions, the hydrotherapy group, as measured using the GMFM-88 scale, showed greater improvement than those undergoing conventional rehabilitation. This improvement is likely due to the buoyancy and support provided by water, which simplifies movement execution. Moreover, the novelty and engaging nature of water activities increase children’s willingness to participate in active movement, thus enhancing their gross motor functions and mitigating issues associated with insufficient physical activity [30,42–44]. These findings are consistent with previous systematic reviews. Pauluka et al. conducted a meta-analysis and found that aquatic physical therapy resulted in significantly higher GMFM scores compared to land-based interventions, although the overall quality of evidence was low due to methodological limitations [17]. Similarly, Roostaei et al. reported within-group improvements in gross motor functions following aquatic interventions, including activities like standing and walking [19]. Faccioli et al. further supported the inclusion of hydrotherapy as a complementary approach to task-oriented physical therapy, emphasizing its motivational and participatory nature [18]. However, Tapia et al. noted that while Halliwick-based aquatic therapy showed potential benefits in gross motor and social domains, the current body of evidence is limited by low methodological rigor [15]. In contrast to earlier reviews focusing on either specific aquatic methods or isolated outcomes, our study contributes by directly comparing hydrotherapy with conventional rehabilitation using standardized outcome measures, thus strengthening the evidence base for clinical decision-making. Nevertheless, moderate heterogeneity (I^2^ = 59.5%) was observed in the meta-analysis, which may be attributed to differences in participant age, intervention duration, and the content of hydrotherapy programs. These factors suggest that such variability may influence the consistency of hydrotherapy’s effects across studies.

As for fine motor functions, enhancements may derive from the multi-sensory stimulation and coordination demands of hydrotherapy, which aid in developing fine motor control. A study by Luo et al. demonstrated that hydrotherapy, combined with conventional training, significantly improved children’s Fine Motor functions scores [32], corroborating the findings of this review. Additionally, research by Zhang Juan et al. suggested that fine motor functions are indicative of neurological coordination, whereas gross motor functions involve both muscular and neurological coordination. Conventional rehabilitation also targets neurological coordination. Although most systematic reviews on aquatic therapy have focused on gross motor outcomes, some also acknowledge its potential benefits for fine motor and coordination functions. For instance, Tapia et al. noted improvements in coordination and social interaction following Halliwick-based aquatic therapy in children with cerebral palsy, though specific fine motor outcomes were not separately measured. Similarly, van ‘t Hooft et al. reported enhanced motor coordination, including object control and manipulation functions, in aquatic interventions among children with neurodevelopmental disorders, supporting the idea that water-based environments may implicitly train fine motor pathways through dynamic resistance and proprioceptive input. While evidence is still limited, these findings suggest a potential role of hydrotherapy in improving fine motor performance, especially when paired with task-specific goals. It is worth noting that this group of studies exhibited a moderate level of heterogeneity (I^2^ = 46.4%). Although all studies employed the same fine motor assessment tool, this heterogeneity may still be attributed to differences in participant age, types of cerebral palsy, and the intensity of interventions.

Badawy et al. utilized the Biodex balance testing system to analyze the impact of hydrotherapy on balance in children and adolescents with cerebral palsy [27]. Following 12 weeks of aquatic exercise therapy, the treatment group demonstrated significantly enhanced balance parameters compared to the conventional training group. Zhang Juan employed the DK-PHY balance detection training system [39], and after three months, the hydrotherapy group showed notably lower balance indicator scores than the conventional training group, suggesting improved stability in standing both with eyes open and closed, as well as reduced speed and distance of lateral movements. However, research by Sung-Hoon Kang et al. and Zhao et al. found no significant differences in balance improvements between the hydrotherapy group and conventional training groups [33,38]. These mixed results are echoed in previous reviews. Getz et al. found that several aquatic intervention studies reported improvements in postural control and balance, though overall evidence was limited by low methodological quality [11]. Similarly, Tapia et al. noted that Halliwick-based hydrotherapy may enhance postural stability in children with cerebral palsy, but balance was often a secondary outcome and inconsistently assessed across studies [15]. These findings suggest potential benefits of hydrotherapy for balance, though more robust evidence is needed. It is worth noting that this group of studies exhibited high heterogeneity (I^2^ = 80.7%). Analysis indicated that heterogeneity may stem from differences in assessment tools, variations in intervention design, and inconsistencies in the age distribution of participants. Sensitivity analysis revealed that the study by Badawy et al. had a significant impact on heterogeneity, suggesting that the results should be interpreted with caution [26].

An analysis involving four studies [26,33,37,41] assessed hydrotherapy’s impact on muscle tone, utilizing the Modified Ashworth Scale (MAS). The results indicated that the hydrotherapy group achieved better MAS scores than the conventional training group, although the overall statistical significance of hydrotherapy’s effect on muscle tone was not established, but a trend favored the hydrotherapy group. Although systematic reviews on aquatic therapy rarely focus specifically on muscle tone, some have noted potential benefits. For example, Tapia et al. observed improvements in mobility and comfort with Halliwick-based therapy, though spasticity was not directly measured [15]. Similarly, Faccioli et al. suggested hydrotherapy as a complementary approach following spasticity treatments, but evidence for its direct effect on tone remains limited [18]. This meta-analysis revealed a moderate level of heterogeneity (I^2^ = 58.2%), which may be attributed to differences across studies in intervention frequency, treatment duration, the proportion of participants with spastic cerebral palsy, and the inherent subjectivity of the MAS assessment. These findings highlight the need for greater standardization of intervention protocols and consistency in outcome evaluation in future research to enhance the comparability and interpretability of results.

The study acknowledges several limitations, particularly regarding the basic characteristics of the included cerebral palsy patients. Participants vary widely in age, and the distribution of different cerebral palsy types is uneven. Most subjects are diagnosed with spastic cerebral palsy, reflecting its prevalence, which accounts for approximately 70%−75% of all cerebral palsy cases [45]. Additionally, there is variability in the Gross Motor Function Classification System (GMFCS) levels among participants. Recent trends show a shift towards more severe cerebral palsy cases. Children and adolescents with more severe symptoms often derive greater benefits from aquatic therapy, as it allows them freedom of movement in water, enhancing their physical and psychosocial well-being. Future research should focus on this subgroup, and more comprehensive studies on aquatic therapy should be undertaken [46], considering various ages, types, and GMFCS levels of cerebral palsy patients.

The study’s limitations include a. Inclusion of only published literature, potentially introducing language bias. b. The nature of rehabilitation treatments precludes blinding of subjects and therapists; most studies lacked evaluator blinding, and many failed to describe allocation concealment clearly, which could lead to bias. c. The study subjects included diverse Cerebral palsy types and varied in age, sex, clinical classification, and GMFCS levels, potentially affecting outcome measures due to differences in disease type and severity. d. Absence of a standardized measurement instrument or scale across the studies. e. The overall sample size is small, and there is a lack of multi-center, large sample studies. f. Specific intervention methods varied, including differences in exercise types, intensity, duration, and frequency, which complicates detailed analysis and may introduce bias. g. Limited long-term follow-up data, hindering the assessment of long-term efficacy. In future aquatic therapy for cerebral palsy, a greater emphasis on precision and standardization is necessary [47]. Improving the methodological quality of clinical trials, increasing sample sizes, extending follow-up periods, refining exercise prescriptions, ensuring evaluator blinding, and standardizing assessment tools are crucial steps forward.

## Conclusion

This study conducted a systematic review and meta-analysis to evaluate the effects of hydrotherapy on motor functions in children and adolescents with cerebral palsy. The results demonstrated that hydrotherapy was significantly more effective than conventional care in improving gross motor functions, with consistent benefits observed across different age groups and in interventions lasting more than 10 weeks. Hydrotherapy also showed a positive trend in enhancing fine motor functions. However, no statistically significant improvements were found in balance or muscle tone. Although some outcomes did not reach statistical significance, the overall trend supports its potential as a beneficial rehabilitation intervention. However, the methodological quality of the studies included varied, with some presenting risks of bias, and the certainty of evidence for several outcomes was rated as moderate to low according to the GRADE assessment. Therefore, the findings should be interpreted with caution. Future research should focus on high-quality, large-scale, and well-designed trials with extended follow-up periods to further validate the effectiveness of hydrotherapy and enhance the reliability and applicability of current evidence.

## Supporting information

S1 File Search strategy. (DOCX)

S1 Table GRADE Assessment. (DOCX)

S1 Checklist PRISMA checklist. (DOCX)

S1 Data Reasons for excluded studies. (DOC)

S2 Data Availability statement. (DOCX)
