# Supplementary material for: The effects of hydrotherapy on athletic ability in children with cerebral palsy: A systematic review and meta-analysis
Source: PLoS One. 2025 Jun 10;20(6):e0325517. doi: 10.1371/journal.pone.0325517 (PMC12151372; doi:10.1371/journal.pone.0325517)
Supplement: S1 File — (DOCX) [file pone.0325517.s001.docx]

**S1 Search strategy**

**PubMed Search Strategy**

Search: ((((((((((((((((((((((((((Whirlpool Baths[Title/Abstract]) OR (Baths, Whirlpool[Title/Abstract])) OR (Bath, Whirlpool[Title/Abstract])) OR (Whirlpool Bath[Title/Abstract])) OR (Watsu Therapy[Title/Abstract])) OR (Therapy, Watsu[Title/Abstract])) OR (WaterShiatsu[Title/Abstract])) OR (Water Shiatsu[Title/Abstract])) OR (Shiatsu, Water[Title/Abstract])) OR (Aquatic Therapy[Title/Abstract])) OR (Therapy, Aquatic[Title/Abstract])) OR (Pool Therapy[Title/Abstract])) OR (Therapy, Pool[Title/Abstract])) OR (Ai Chi Therapy[Title/Abstract])) OR (Therapies, Ai Chi[Title/Abstract])) OR (Therapy, Ai Chi[Title/Abstract])) OR (Water Tai Chi Therapy[Title/Abstract])) OR (Aquatic Exercise Therapy[Title/Abstract])) OR (Exercise Therapy, Aquatic[Title/Abstract])) OR (Therapy, Aquatic Exercise[Title/Abstract])) OR (Water Exercise Therapy[Title/Abstract])) OR (Exercise Therapy, Water[Title/Abstract])) OR (Therapy, Water Exercise[Title/Abstract])) OR (Halliwick[Title/Abstract])) OR (swimming exercises[Title/Abstract])) OR ("Hydrotherapy"[Mesh])) AND (((((((((((((((((((((((((((((((((((((((((((CP (Cerebral Palsy[Title/Abstract])) OR (Little Disease[Title/Abstract])) OR (Diplegia, Spastic[Title/Abstract])) OR (Little's Disease[Title/Abstract])) OR (Spastic Diplegia[Title/Abstract])) OR (Diplegias, Spastic[Title/Abstract])) OR (Spastic Diplegias[Title/Abstract])) OR (Cerebral Palsy, Atonic[Title/Abstract])) OR (Atonic Cerebral Palsy[Title/Abstract])) OR (Cerebral Palsy, Hypotonic[Title/Abstract])) OR (Hypotonic Cerebral Palsies[Title/Abstract])) OR (Hypotonic Cerebral Palsy[Title/Abstract])) OR (Cerebral Palsy, Athetoid[Title/Abstract])) OR (Athetoid Cerebral Palsy[Title/Abstract])) OR (Cerebral Palsies, Athetoid[Title/Abstract])) OR (Cerebral Palsies, Athetoid[Title/Abstract])) OR (Cerebral Palsy, Dyskinetic[Title/Abstract])) OR (Cerebral Palsies, Dyskinetic[Title/Abstract])) OR (Dyskinetic Cerebral Palsy[Title/Abstract])) OR (Monoplegic Cerebral Palsy[Title/Abstract])) OR (Cerebral Palsies, Monoplegic[Title/Abstract])) OR (Cerebral Palsy, Monoplegic[Title/Abstract])) OR (Monoplegic Cerebral Palsies[Title/Abstract])) OR (Cerebral Palsy, Quadriplegic, Infantile[Title/Abstract])) OR (Infantile Cerebral Palsy, Quadriplegic[Title/Abstract])) OR (Quadriplegic Infantile Cerebral Palsy[Title/Abstract])) OR (Cerebral Palsy, Congenital[Title/Abstract])) OR (Congenital Cerebral Palsy[Title/Abstract])) OR (Cerebral Palsy, Diplegic, Infantile[Title/Abstract])) OR (Diplegic Infantile Cerebral Palsy[Title/Abstract])) OR (Infantile Cerebral Palsy, Diplegic[Title/Abstract])) OR (Cerebral Palsy, Dystonic-Rigid[Title/Abstract])) OR (Cerebral Palsies, Dystonic-Rigid[Title/Abstract])) OR (Dystonic-Rigid Cerebral Palsies[Title/Abstract])) OR (Dystonic-Rigid Cerebral Palsy[Title/Abstract])) OR (Cerebral Palsy, Mixed[Title/Abstract])) OR (Mixed Cerebral Palsies[Title/Abstract])) OR (Cerebral Palsy, Rolandic Type[Title/Abstract])) OR (Cerebral Palsy, Spastic[Title/Abstract])) OR (Spastic Cerebral Palsies[Title/Abstract])) OR (Infantile Cerebral Palsy, Monoplegic[Title/Abstract])) OR (Monoplegic Infantile Cerebral Palsy[Title/Abstract])) OR (Cerebral Palsy))

**Embase Search Strategy**

'CP (Cerebral Palsy)' or 'Little Disease' or 'Diplegia, Spastic' or 'Little's Disease' or 'Spastic Diplegia' or 'Diplegias, Spastic' or 'Spastic Diplegias' or 'Cerebral Palsy, Atonic' or 'Atonic Cerebral Palsy' or 'Cerebral Palsy, Hypotonic' or 'Hypotonic Cerebral Palsies' or 'Hypotonic Cerebral Palsy' or 'Cerebral Palsy, Athetoid' or 'Athetoid Cerebral Palsy' or 'Cerebral Palsies, Athetoid' or 'Cerebral Palsy, Dyskinetic' or 'Cerebral Palsies, Dyskinetic' or 'Dyskinetic Cerebral Palsy' or 'Monoplegic Cerebral Palsy' or 'Cerebral Palsies, Monoplegic' or 'Cerebral Palsy, Monoplegic' or 'Monoplegic Cerebral Palsies' or 'Cerebral Palsy, Quadriplegic, Infantile' or 'Infantile Cerebral Palsy, Quadriplegic' or 'Quadriplegic Infantile Cerebral Palsy' or 'Cerebral Palsy, Congenital' or 'Congenital Cerebral Palsy' or 'Cerebral Palsy, Diplegic, Infantile' or 'Diplegic Infantile Cerebral Palsy' or 'Infantile Cerebral Palsy, Diplegic' or 'Cerebral Palsy, Dystonic-Rigid' or 'Cerebral Palsies, Dystonic-Rigid' or 'Cerebral Palsy, Dystonic Rigid' or 'Dystonic-Rigid Cerebral Palsies' or 'Dystonic-Rigid Cerebral Palsy' or 'Cerebral Palsy, Mixed' or 'Mixed Cerebral Palsies' or 'Mixed Cerebral Palsy' or 'Cerebral Palsy, Rolandic Type' or 'Rolandic Type Cerebral Palsy' or 'Cerebral Palsy, Spastic' or 'Spastic Cerebral Palsies' or 'Spastic Cerebral Palsy' or 'Cerebral Palsy, Monoplegic, Infantile' or 'Infantile Cerebral Palsy, Monoplegic' or 'Monoplegic Infantile Cerebral Palsy'

**Web of Science Basic Search Strategy**

ID Search Hits

#1 MeSH descriptor: [Hydrotherapy] explode all trees 1924

#2 (Whirlpool Baths):ti,ab,kw or (Baths, Whirlpool):ti,ab,kw or (Bath, Whirlpool):ti,ab,kw or (Whirlpool Bath):ti,ab,kw or (Watsu Therapy):ti,ab,kw or (Therapy, Watsu):ti,ab,kw or (WaterShiatsu):ti,ab,kw or (Water Shiatsu):ti,ab,kw or (Shiatsu, Water):ti,ab,kw or (Aquatic Therapy):ti,ab,kw or (Therapy, Aquatic):ti,ab,kw or (Pool Therapy):ti,ab,kw or (Therapy, Pool):ti,ab,kw or (Ai Chi Therapy):ti,ab,kw or (Therapies, Ai Chi):ti,ab,kw or (Therapy, Ai Chi):ti,ab,kw or (Water Tai Chi Therapy):ti,ab,kw or (Aquatic Exercise Therapy):ti,ab,kw or (Exercise Therapy, Aquatic):ti,ab,kw or (Therapy, Aquatic Exercise):ti,ab,kw or (Water Exercise Therapy):ti,ab,kw or (Exercise Therapy, Water):ti,ab,kw or (Therapy, Water Exercise):ti,ab,kw or (Aquatic Therapy):ti,ab,kw or (swimming exercises) 3681

#3 #1 OR #2 5485

#4 Cerebral Palsy 6105

#5 (CP (Cerebral Palsy)):ti,ab,kw or (Little Disease):ti,ab,kw or (Diplegia, Spastic):ti,ab,kw or (Little's Disease):ti,ab,kw or (Spastic Diplegia):ti,ab,kw or (Diplegias, Spastic):ti,ab,kw or (Spastic Diplegias):ti,ab,kw or (Cerebral Palsy, Atonic):ti,ab,kw or (Atonic Cerebral Palsy):ti,ab,kw or (Cerebral Palsy, Hypotonic):ti,ab,kw or (Hypotonic Cerebral Palsies):ti,ab,kw or (Hypotonic Cerebral Palsy):ti,ab,kw or (Cerebral Palsy, Athetoid):ti,ab,kw or (Athetoid Cerebral Palsy):ti,ab,kw or (Cerebral Palsies, Athetoid):ti,ab,kw or (Cerebral Palsy, Dyskinetic):ti,ab,kw or (Cerebral Palsies, Dyskinetic):ti,ab,kw or (Dyskinetic Cerebral Palsy):ti,ab,kw or (Monoplegic Cerebral Palsy):ti,ab,kw or (Cerebral Palsies, Monoplegic):ti,ab,kw or (Cerebral Palsy, Monoplegic):ti,ab,kw or (Monoplegic Cerebral Palsies):ti,ab,kw or (Cerebral Palsy, Quadriplegic, Infantile):ti,ab,kw or (Infantile Cerebral Palsy, Quadriplegic):ti,ab,kw or (Quadriplegic Infantile Cerebral Palsy):ti,ab,kw or (Cerebral Palsy, Congenital):ti,ab,kw or (Congenital Cerebral Palsy):ti,ab,kw or (Cerebral Palsy, Diplegic, Infantile):ti,ab,kw or (Diplegic Infantile Cerebral Palsy):ti,ab,kw or (Infantile Cerebral Palsy, Diplegic):ti,ab,kw or (Cerebral Palsy, Dystonic-Rigid):ti,ab,kw or (Cerebral Palsies, Dystonic-Rigid):ti,ab,kw or (Cerebral Palsy, Dystonic Rigid):ti,ab,kw or (Dystonic-Rigid Cerebral Palsies):ti,ab,kw or (Dystonic-Rigid Cerebral Palsy):ti,ab,kw or (Cerebral Palsy, Mixed):ti,ab,kw or (Mixed Cerebral Palsies):ti,ab,kw or (Mixed Cerebral Palsy):ti,ab,kw or (Cerebral Palsy, Rolandic Type):ti,ab,kw or (Rolandic Type Cerebral Palsy):ti,ab,kw or (Cerebral Palsy, Spastic):ti,ab,kw or (Spastic Cerebral Palsies):ti,ab,kw or (Spastic Cerebral Palsy):ti,ab,kw or (Cerebral Palsy, Monoplegic, Infantile):ti,ab,kw or (Infantile Cerebral Palsy, Monoplegic):ti,ab,kw or (Monoplegic Infantile Cerebral Palsy) 12679

#6 #4 OR #5 15623

#7 Child 217570

#8 Children 217570

#9 #3 and #6 and #7 and #8 85

**Cochrane Library Search Strategy**

****Searches:****

| Searches: | MeSH descriptor: [Hydrotherapy] explode all trees | 1922 |
| --- | --- | --- |
| #2 | (Whirlpool Baths):ti,ab,kw or (Baths, Whirlpool):ti,ab,kw or (Bath, Whirlpool):ti,ab,kw or (Whirlpool Bath):ti,ab,kw or (Watsu Therapy):ti,ab,kw or (Therapy, Watsu):ti,ab,kw or (WaterShiatsu):ti,ab,kw or (Water Shiatsu):ti,ab,kw or (Shiatsu, Water):ti,ab,kw or (Aquatic Therapy):ti,ab,kw or (Therapy, Aquatic):ti,ab,kw or (Pool Therapy):ti,ab,kw or (Therapy, Pool):ti,ab,kw or (Ai Chi Therapy):ti,ab,kw or (Therapies, Ai Chi):ti,ab,kw or (Therapy, Ai Chi):ti,ab,kw or (Water Tai Chi Therapy):ti,ab,kw or (Aquatic Exercise Therapy):ti,ab,kw or (Exercise Therapy, Aquatic):ti,ab,kw or (Therapy, Aquatic Exercise):ti,ab,kw or (Water Exercise Therapy):ti,ab,kw or (Exercise Therapy, Water):ti,ab,kw or (Therapy, Water Exercise):ti,ab,kw or (Aquatic Therapy):ti,ab,kw or (swimming exercises) | 3659 |
| #3 | #1 OR #2 | 5462 |
| #4 | Cerebral Palsy | 6071 |
| #5 | (CP (Cerebral Palsy)):ti,ab,kw or (Little Disease):ti,ab,kw or (Diplegia, Spastic):ti,ab,kw or (Little's Disease):ti,ab,kw or (Spastic Diplegia):ti,ab,kw or (Diplegias, Spastic):ti,ab,kw or (Spastic Diplegias):ti,ab,kw or (Cerebral Palsy, Atonic):ti,ab,kw or (Atonic Cerebral Palsy):ti,ab,kw or (Cerebral Palsy, Hypotonic):ti,ab,kw or (Hypotonic Cerebral Palsies):ti,ab,kw or (Hypotonic Cerebral Palsy):ti,ab,kw or (Cerebral Palsy, Athetoid):ti,ab,kw or (Athetoid Cerebral Palsy):ti,ab,kw or (Cerebral Palsies, Athetoid):ti,ab,kw or (Cerebral Palsy, Dyskinetic):ti,ab,kw or (Cerebral Palsies, Dyskinetic):ti,ab,kw or (Dyskinetic Cerebral Palsy):ti,ab,kw or (Monoplegic Cerebral Palsy):ti,ab,kw or (Cerebral Palsies, Monoplegic):ti,ab,kw or (Cerebral Palsy, Monoplegic):ti,ab,kw or (Monoplegic Cerebral Palsies):ti,ab,kw or (Cerebral Palsy, Quadriplegic, Infantile):ti,ab,kw or (Infantile Cerebral Palsy, Quadriplegic):ti,ab,kw or (Quadriplegic Infantile Cerebral Palsy):ti,ab,kw or (Cerebral Palsy, Congenital):ti,ab,kw or (Congenital Cerebral Palsy):ti,ab,kw or (Cerebral Palsy, Diplegic, Infantile):ti,ab,kw or (Diplegic Infantile Cerebral Palsy):ti,ab,kw or (Infantile Cerebral Palsy, Diplegic):ti,ab,kw or (Cerebral Palsy, Dystonic-Rigid):ti,ab,kw or (Cerebral Palsies, Dystonic-Rigid):ti,ab,kw or (Cerebral Palsy, Dystonic Rigid):ti,ab,kw or (Dystonic-Rigid Cerebral Palsies):ti,ab,kw or (Dystonic-Rigid Cerebral Palsy):ti,ab,kw or (Cerebral Palsy, Mixed):ti,ab,kw or (Mixed Cerebral Palsies):ti,ab,kw or (Mixed Cerebral Palsy):ti,ab,kw or (Cerebral Palsy, Rolandic Type):ti,ab,kw or (Rolandic Type Cerebral Palsy):ti,ab,kw or (Cerebral Palsy, Spastic):ti,ab,kw or (Spastic Cerebral Palsies):ti,ab,kw or (Spastic Cerebral Palsy):ti,ab,kw or (Cerebral Palsy, Monoplegic, Infantile):ti,ab,kw or (Infantile Cerebral Palsy, Monoplegic):ti,ab,kw or (Monoplegic Infantile Cerebral Palsy) | 12628 |
| #6 | #4 OR #5 | 15559 |
| #7 | #3 AND #6 | 147 |
| #8 | Child | 216778 |
| #9 | Children | 216778 |
| #10 | #8 and #9 and #7 | 82 |

**CNKI Search Strategy**

条件： ( ( ( ( ( 主题%= 水疗+浴法+浴疗+浸浴疗法+早期水疗 or 主题%= 水療+浴法+浴療+浸浴療法+早期水療 or 题名%= 水疗+浴法+浴疗+浸浴疗法+早期水疗 or 题名%= 水療+浴法+浴療+浸浴療法+早期水療 ) OR ( 主题%='水中运动' or 主题%='水中運動' or 题名%='水中运动' or 题名%='水中運動' ) ) OR ( 主题%='游泳' or 题名%='游泳' ) ) AND ( ( 主题%= 脑瘫+痉挛型脑瘫+痉挛型脑性瘫痪+痉挛型四肢瘫+痉挛型双瘫+偏瘫 or 主题%= 腦癱+痙攣型腦癱+痙攣型腦性癱瘓+痙攣型四肢癱+痙攣型雙癱+偏癱 or 题名%= 脑瘫+痉挛型脑瘫+痉挛型脑性瘫痪+痉挛型四肢瘫+痉挛型双瘫+偏瘫 or 题名%= 腦癱+痙攣型腦癱+痙攣型腦性癱瘓+痙攣型四肢癱+痙攣型雙癱+偏癱 ) OR ( 主题%= LITTLE病+运动障碍+大脑麻痹+大脑瘫 or 主题%= LITTLE病+運動障礙+大腦麻痹+大腦癱 or 题名%= LITTLE病+运动障碍+大脑麻痹+大脑瘫 or 题名%= LITTLE病+運動障礙+大腦麻痹+大腦癱 ) ) ) AND ( ( 主题%= 儿童+学龄前儿童 or 主题%= 兒童+學齡前兒童 or 题名%= 儿童+学龄前儿童 or 题名%= 兒童+學齡前兒童 ) OR ( 主题%='青少年' or 题名%='青少年' ) ) )

**Wanfang Database Search Strategy**

主题:(水疗 or 浴疗 or 游泳 or 水中运动 or 温泉 or 水疗池 ) and 主题:(脑瘫 or 运动障碍 or 痉挛型脑瘫 or 神经系统疾病 or 运动发育迟缓 or 脑损伤 or 脑部缺氧) and 主题:(儿童 or 青少年) and 主题:(运动能力 or 运动技能 or 肌张力 or 粗大运动 or 平衡 or 精细运动 or 速度 or 运动 or 耐力 or 灵敏)

**VIP Database Search Strategy**

[(((((((题名或关键词=水疗 OR 题名或关键词=水中运动) OR 题名或关键词=游泳) OR 题名或关键词=浴疗) OR 题名或关键词=泳池) AND ((((((((((((((题名或关键词=脑瘫 OR 题名或关键词=cerebral palsies) OR 题名或关键词=cerebral palsy) OR 题名或关键词=痉挛型脑瘫) OR 题名或关键词=小儿脑瘫) OR 题名或关键词=痉挛型小儿脑性瘫痪) OR 题名或关键词=痉挛型脑性瘫痪) OR 题名或关键词=痉挛性脑性瘫痪) OR 题名或关键词=儿童脑性瘫痪) OR 题名或关键词=大脑性瘫痪) OR 题名或关键词=脑性瘫疾) OR 题名或关键词=痉挛性脑瘫) OR 题名或关键词=脑性瘫痪) OR 题名或关键词=小儿脑性瘫痪) OR 题名或关键词=脑型瘫痪)) AND ((((((((((题名或关键词=运动能力 OR 题名或关键词=exercise ability) OR 题名或关键词=motion ability) OR 题名或关键词=motor ability) OR 题名或关键词=sports ability) OR 题名或关键词=灵敏) OR 题名或关键词=耐力) OR 题名或关键词=肌张力) OR 题名或关键词=精细运动) OR 题名或关键词=粗大运动) OR 题名或关键词=运动)) AND (摘要=随机 OR 摘要=随机对照))](https://qikan.cqvip.com/Qikan/search/index?LngMySearHistoryIdGuid=17242e62-77fa-482a-8d6a-aa9bab5e533c&from=Qikan_Article_History)
