# Supplementary material for: The effects of hydrotherapy on athletic ability in children with cerebral palsy: A systematic review and meta-analysis
Source: PLoS One. 2025 Jun 10;20(6):e0325517. doi: 10.1371/journal.pone.0325517 (PMC12151372; doi:10.1371/journal.pone.0325517)
Supplement: S1 Table — (DOCX) [file pone.0325517.s002.docx]

**S2 Table. GRADE Assessment**

| **Outcomes** | **Study**  **Design** | **Risk of Bias** | **Inconsistency** | **Indirectness** | **Imprecision** | **Other**  **Considerations** | **Effect (95% CI)** | **I^2^** | **Quality** |
| --- | --- | --- | --- | --- | --- | --- | --- | --- | --- |
| Gross motor functions | 12 RCTs | Serious | Serious | No | No | No | SMD 0.41 (0.15 to 0.68) | 59.5% | Low |
| Treat period≤ 10 weeks | 4 RCTs | Serious | Serious | No | No | Serious | SMD 0.14 (-0.26 to 0.53) | 35.6% | Very low |
| Treat period> 10 weeks | 8 RCTs | Serious | Serious | No | No | No | SMD 0.48 (0.31 to 0.66) | 65.1% | Low |
| Age≤ 6 years | 6 RCTs | Serious | Serious | No | No | No | SMD 0.42 (0.16 to 0.68) | 38.2% | Low |
| Age> 6 years | 6 RCTs | Serious | Serious | No | No | No | SMD 0.43 (0.22 to 0.63) | 73.8% | Low |
| Fine motor functions | 2 RCTs | Serious | No | No | Serious | Serious | SMD 0.78 (0.46 to 1.10) | 46.4% | Very low |
| Balance | 4 RCTs | Serious | Serious | No | Serious | No | SMD 0.64 (-0.05 to 1.34) | 80.7% | Very low |
| Muscle tone | 4 RCTs | Serious | Serious | No | Serious | No | SMD -0.45 (-0.98 to 0.07) | 58.2% | Very low |

RCTs, randomized controlled trials. MD, mean difference. SMD, standardized mean difference.
