# Supplementary material for: The effects of hydrotherapy on athletic ability in children with cerebral palsy: A systematic review and meta-analysis
Source: PLoS One. 2025 Jun 10;20(6):e0325517. doi: 10.1371/journal.pone.0325517 (PMC12151372; doi:10.1371/journal.pone.0325517)
Supplement: S1 Data — (DOC) [file pone.0325517.s004.doc]

**S4 Reasons for excluded studies**

| **Studies identified in the literature search (N = 543)** | |
| --- | --- |
| **Reasons for exclusion** | **Citations** |
| Duplicate records removed (n = 97) | 1. Ostojic K, Paget S, Kyriagis M, et al. Acute and chronic pain in children and adolescents with cerebral palsy: prevalence, interference, and management[J]. Archives of physical medicine and rehabilitation, 2020, 101(2): 213-219. 2. Schitter A M, Fleckenstein J, Frei P, et al. Applications, indications, and effects of passive hydrotherapy WATSU (WaterShiatsu)—A systematic review and meta-analysis[J]. PloS one, 2020, 15(3): e0229705. 3. Pauluka E, Ceolin L S, Fontanela L C, et al. Aquatic Compared With Land‐Based Exercises on Gross Motor Function of Children/Adolescents With Cerebral Palsy: A Systematic Review With Meta‐Analysis[J]. Child: Care, Health and Development, 2025, 51(1): e70023. 4. Pauluka E, Ceolin L S, Fontanela L C, et al. Aquatic Compared With Land‐Based Exercises on Gross Motor Function of Children/Adolescents With Cerebral Palsy: A Systematic Review With Meta‐Analysis[J]. Child: Care, Health and Development, 2025, 51(1): e70023. 5. Fragala-Pinkham M A, Dumas H M, Barlow C A, et al. An aquatic physical therapy program at a pediatric rehabilitation hospital: a case series[J]. Pediatric Physical Therapy, 2009, 21(1): 68-78. 6. Xiang A, Fu Y, Wang C, et al. Aquatic therapy for spastic cerebral palsy: a scoping review[J]. European Journal of Medical Research, 2024, 29(1): 569. 7. Xiang A, Fu Y, Wang C, et al. Aquatic therapy for spastic cerebral palsy: a scoping review[J]. European Journal of Medical Research, 2024, 29(1): 569. 8. Ogonowska-Slodownik A, Jakobowicz O, Alexander L, et al. Aquatic Therapy in Children and Adolescents with Disabilities: A Scoping Review[J]. Children, 2024, 11(11): 1404. 9. Becker B E. Aquatic therapy in contemporary neurorehabilitation: an update[J]. Pm&r, 2020, 12(12): 1251-1259. 10. Becker B E. Aquatic therapy in contemporary neurorehabilitation: an update[J]. Pm&r, 2020, 12(12): 1251-1259. 11. Trevelyan J. Aquatots[J]. Nursing Times, 1990, 86(15): 46-47. 12. Chen X L, Yu L P, Zhu Y, et al. Combined effect of hydrotherapy and transcranial direct-current stimulation on children with cerebral palsy: A protocol for a randomized controlled trial[J]. Medicine, 2021, 100(49): e27962. 13. Chen X L, Yu L P, Zhu Y, et al. Combined effect of hydrotherapy and transcranial direct-current stimulation on children with cerebral palsy: A protocol for a randomized controlled trial[J]. Medicine, 2021, 100(49): e27962. 14. Chen X L, Yu L P, Zhu Y, et al. Combined effect of hydrotherapy and transcranial direct-current stimulation on children with cerebral palsy: A protocol for a randomized controlled trial[J]. Medicine, 2021, 100(49): e27962. 15. Mostafa A M A, El-Negmy E H, Abd El-Maksoud G M, et al. Effect of aquatic therapy on head control in cerebral palsy children[J]. Current Pediatric Research, 2021, 25(12): 1142-1149. 16. Isshiki Y. Effect of oral cleansing in cerebral palsied children (application of a water jet device)[J]. The Bulletin of Tokyo Dental College, 1970, 11(2): 121-131. 17. Warnier N, Lambregts S, Port I V D. Effect of virtual reality therapy on balance and walking in children with cerebral palsy: a systematic review[J]. Developmental neurorehabilitation, 2020, 23(8): 502-518. 18. Tapia C, Constanzo J, González V, et al. The effectiveness of aquatic therapy based on the Halliwick concept in children with cerebral palsy: A systematic review[J]. Developmental Neurorehabilitation, 2023, 26(6-7): 371-376. 19. Tapia C, Constanzo J, González V, et al. The effectiveness of aquatic therapy based on the Halliwick concept in children with cerebral palsy: A systematic review[J]. Developmental Neurorehabilitation, 2023, 26(6-7): 371-376. 20. Chandolias K, Tsounia E A, Strimpakos N, et al. The effectiveness of the Halliwick concept hydrotherapy and Bobath (Neurodevelopmental Treatment-NDT) in the treatment of children with cerebral palsy–A randomized controlled trial[J]. Brain Disorders, 2025: 100213. 21. Chandolias K, Tsounia E A, Strimpakos N, et al. The effectiveness of the Halliwick concept hydrotherapy and Bobath (Neurodevelopmental Treatment-NDT) in the treatment of children with cerebral palsy–A randomized controlled trial[J]. Brain Disorders, 2025: 100213. 22. Jorgić B, Dimitrijević L, Aleksandrović M, et al. Effects of 12-week aquatic exercises on gross motor function, swimming skills and walking ability in children with cerebral palsy[J]. Minerva pediatrics, 2024, 76(2): 149-160. 23. Jorgić B, Dimitrijević L, Aleksandrović M, et al. Effects of 12-week aquatic exercises on gross motor function, swimming skills and walking ability in children with cerebral palsy[J]. Minerva pediatrics, 2024, 76(2): 149-160. 24. Retarekar R, Fragala-Pinkham M A, Townsend E L. Effects of aquatic aerobic exercise for a child with cerebral palsy: single-subject design[J]. Pediatric physical therapy, 2009, 21(4): 336-344. 25. Roostaei M, Baharlouei H, Azadi H, et al. Effects of aquatic intervention on gross motor skills in children with cerebral palsy: a systematic review[J]. Physical & occupational therapy in pediatrics, 2017, 37(5): 496-515. 26. Roostaei M, Baharlouei H, Azadi H, et al. Effects of aquatic intervention on gross motor skills in children with cerebral palsy: a systematic review[J]. Physical & occupational therapy in pediatrics, 2017, 37(5): 496-515. 27. Getz M, Hutzler Y, Vermeer A. Effects of aquatic interventions in children with neuromotor impairments: a systematic review of the literature[J]. Clinical rehabilitation, 2006, 20(11): 927-936. 28. Kokavec M, Fristáková M. Efficacy of antiseptics in the prevention of post-operative infections of the proximal femur, hip and pelvis regions in orthopedic pediatric patients. Analysis of the first results[J]. Acta chirurgiae orthopaedicae et traumatologiae Cechoslovaca, 2008, 75(2): 106-109. 29. Abdelaal A A M, Atia D. Efficacy of aquatic exercise on pulmonary function and aquatic skills performance in older children with cerebral palsy. Randomised controlled study[J]. Physiotherapy Quarterly, 2023, 31(4): 81-86. 30. Duncan B, Shen K, Zou L P, et al. Evaluating intense rehabilitative therapies with and without acupuncture for children with cerebral palsy: a randomized controlled trial[J]. Archives of Physical Medicine and Rehabilitation, 2012, 93(5): 808-815. 31. Duncan B, Shen K, Zou L P, et al. Evaluating intense rehabilitative therapies with and without acupuncture for children with cerebral palsy: a randomized controlled trial[J]. Archives of Physical Medicine and Rehabilitation, 2012, 93(5): 808-815. 32. Duncan B, Shen K, Zou L P, et al. Evaluating intense rehabilitative therapies with and without acupuncture for children with cerebral palsy: a randomized controlled trial[J]. Archives of Physical Medicine and Rehabilitation, 2012, 93(5): 808-815. 33. Franki I, Desloovere K, De Cat J, et al. The evidence-base for conceptual approaches and additional therapies targeting lower limb function in children with in Cerebral Palsy: A systematic review using the ICF as a framework[J]. Journal of rehabilitation medicine, 2012, 44(5): 396-405. 34. Franki I, Desloovere K, De Cat J, et al. The evidence-base for conceptual approaches and additional therapies targeting lower limb function in children with in Cerebral Palsy: A systematic review using the ICF as a framework[J]. Journal of rehabilitation medicine, 2012, 44(5): 396-405. 35. Franki I, Desloovere K, De Cat J, et al. The evidence-base for conceptual approaches and additional therapies targeting lower limb function in children with in Cerebral Palsy: A systematic review using the ICF as a framework[J]. Journal of rehabilitation medicine, 2012, 44(5): 396-405. 36. Faccioli S, Pagliano E, Ferrari A, et al. Evidence-based management and motor rehabilitation of cerebral palsy children and adolescents: A systematic review[J]. Frontiers in Neurology, 2023, 14: 1171224. 37. Rohn S, Novak Pavlic M, Rosenbaum P. Exploring the use of Halliwick aquatic therapy in the rehabilitation of children with disabilities: A scoping review[J]. Child: care, health and development, 2021, 47(6): 733-743. 38. Rohn S, Novak Pavlic M, Rosenbaum P. Exploring the use of Halliwick aquatic therapy in the rehabilitation of children with disabilities: A scoping review[J]. Child: care, health and development, 2021, 47(6): 733-743. 39. Faccioli S, Cavalagli A, Falocci N, et al. Gait analysis patterns and rehabilitative interventions to improve gait in persons with hereditary spastic paraplegia: a systematic review and meta-analysis[J]. Frontiers in Neurology, 2023, 14: 1256392. 40. Ballaz L, Plamondon S, Lemay M. Group aquatic training improves gait efficiency in adolescents with cerebral palsy[J]. Disability and Rehabilitation, 2011, 33(17-18): 1616-1624. 41. Ballaz L, Plamondon S, Lemay M. Group aquatic training improves gait efficiency in adolescents with cerebral palsy[J]. Disability and Rehabilitation, 2011, 33(17-18): 1616-1624. 42. Ban R. The importance of hydrokinetotherapy in rehabilitation activities for locomotor deficiencies in cerebral palsy[J]. Viata Medicala; Revista de Informare Profesionala si Stiintifica a Cadrelor Medii Sanitare, 1984, 32(5): 105-106. 43. Muñoz-Blanco E, Merino-Andrés J, Aguilar-Soto B, et al. Influence of aquatic therapy in children and youth with cerebral palsy: A qualitative case study in a special education school[J]. International journal of environmental research and public health, 2020, 17(10): 3690. 44. Harris S R. Neurodevelopmental treatment approach for teaching swimming to cerebral palsied children[J]. Physical Therapy, 1978, 58(8): 979-983. 45. Williamson E. No Evidence to Support Aquatic Therapy for Children With Cerebral Palsy, But Do Their Experiences Matter More?[J]. Pediatric Physical Therapy, 2023, 35(4): 386. 46. Lai C J, Liu W Y, Yang T F, et al. Pediatric aquatic therapy on motor function and enjoyment in children diagnosed with cerebral palsy of various motor severities[J]. Journal of child neurology, 2015, 30(2): 200-208. 47. Lai C J, Liu W Y, Yang T F, et al. Pediatric aquatic therapy on motor function and enjoyment in children diagnosed with cerebral palsy of various motor severities[J]. Journal of child neurology, 2015, 30(2): 200-208. 48. Nathanson A T, Young J M J, Young C. Pre-participation medical evaluation for adventure and wilderness watersports[J]. Wilderness & environmental medicine, 2015, 26(4_suppl): 55-62. 49. Nathanson A T, Young J M J, Young C. Pre-participation medical evaluation for adventure and wilderness watersports[J]. Wilderness & environmental medicine, 2015, 26(4_suppl): 55-62. 50. Li F, Huang W, Li H, et al. Rehabilitation effect of aquatic exercise on motor function in children with cerebral palsy: Systematic review of randomized controlled trials[J]. Journal of Back and Musculoskeletal Rehabilitation, 2025: 10538127251313951. 51. Koreniuk S V, Petrova L D. Sanatorium-health resort treatment of children with cerebral palsy in Evpatoria[J]. Pediatriia, 1970, 49(5): 64-66. 52. Koreniuk S V, Petrova L D. Sanatorium-health resort treatment of children with cerebral palsy in Evpatoria[J]. Pediatriia, 1970, 49(5): 64-66. 53. Rogers A, Furler B L, Brinks S, et al. A systematic review of the effectiveness of aerobic exercise interventions for children with cerebral palsy: an AACPDM evidence report[J]. Developmental Medicine & Child Neurology, 2008, 50(11): 808-814. 54. Rogers A, Furler B L, Brinks S, et al. A systematic review of the effectiveness of aerobic exercise interventions for children with cerebral palsy: an AACPDM evidence report[J]. Developmental Medicine & Child Neurology, 2008, 50(11): 808-814. 55. Campbell E D R, Green E A. Treatment of cerebral palsy in the severely subnormal[J]. Rheumatology, 1970, 10(8): 416-420. 56. Naumann K, Kernot J, Parfitt G, et al. Water-based interventions for people with neurological disability, autism, and intellectual disability: A scoping review[J]. Adapted Physical Activity Quarterly, 2021, 38(3): 474-493. 57. Naumann K, Kernot J, Parfitt G, et al. Water-based interventions for people with neurological disability, autism, and intellectual disability: A scoping review[J]. Adapted Physical Activity Quarterly, 2021, 38(3): 474-493. 58. Rogers A, Furler B L, Brinks S, et al. A systematic review of the effectiveness of aerobic exercise interventions for children with cerebral palsy: an AACPDM evidence report[J]. Developmental Medicine & Child Neurology, 2008, 50(11): 808-814. 59. Xiong Youhong, He Xiaohui, Tang Qiaoping, et al. Clinical study on the improvement of walking ability in children with spastic cerebral palsy through MOTOmed intelligent training combined with hydrotherapy. Chinese Journal of Rehabilitation Medicine, 2014, 29(5): 481-482. 60. Hou Xiaohui, Wan Yu, Li Chuyang, et al. The effect of Halliwick technique on gait in school-aged children with spastic cerebral palsy. Chinese Journal of Rehabilitation Medicine, 2010 (9): 870-874. 61. Hou Xiaohui, Wan Yu, Li Chuyang, et al. The effect of Halliwick technique on gait in school-aged children with spastic cerebral palsy. Chinese Journal of Rehabilitation Medicine, 2010 (9): 870-874. 62. Hou Xiaohui, Li Yuhe, Wang Chunyang, et al. The effect of Halliwick technique on balance and gross motor function in school-aged children with cerebral palsy. Chinese Journal of Sports Medicine, 2013, 32(10): 875-880. 63. Liao Ting, Cong Fang, Huang Kunlun. Health and functional benefits of aquatic rehabilitation for disabled children: A systematic review based on ICD-11 and ICF. Chinese Journal of Rehabilitation Theory and Practice, 2023, 29(11): 1286-1295. 64. Luo Wenwen. Analysis of the effect of functional hydrotherapy on muscle strength and motor function in children with spastic cerebral palsy. Chinese Journal of Therapeutic Medicine, 2018, 27(9): 0-0. 65. Luo Wenwen. Analysis of the effect of functional hydrotherapy on muscle strength and motor function in children with spastic cerebral palsy. Chinese Journal of Therapeutic Medicine, 2018, 27(9): 0-0. 66. Wang Guoxiang, Liang Bing, Tao Rong, et al. Evaluation of motor function in children with cerebral palsy based on ICF-CY and hydrotherapy program. Chinese Journal of Rehabilitation Theory and Practice, 2017, 23(2): 146-150. 67. Xie Qiaoling, Liu Zhenhuan. Clinical research progress on early intervention in children with brain injury syndrome. Proceedings of the 19th National Pediatric Chinese and Western Medicine Academic Conference, 2015. 68. Xie Qiaoling, Liu Zhenhuan. Clinical research progress on early intervention in children with brain injury syndrome. Proceedings of the 19th National Pediatric Chinese and Western Medicine Academic Conference, 2015. 69. Hao Wenzhe, Wu Weihong. Characteristics of respiratory function in children with cerebral palsy and the role of hydrotherapy. Chinese Journal of Rehabilitation Theory and Practice, 2008, 14(10): 948-950. 70. Yang Xialin, Tao Xinping, Yu Xiaolan. The "three-level" standardized plan for cerebral palsy rehabilitation: Insights from the rehabilitation practices of children with cerebral palsy at the Pingliang Children's Welfare Institute in Gansu Province. Social Welfare, 2012 (9): 40-41. 71. Tang Jiulai. Recent Advances in Rehabilitation Concepts and Techniques for Cerebral Palsy. Chinese Journal of Children's Health Care, 2017, 25(5): 433. 72. Song Meiju, Liu Jing, Meng Xiaohui. The Therapeutic Effect of Hydrotherapy on Spastic Cerebral Palsy Children. Chinese Journal of Practical Neurological Diseases, 2006, 9(5): 145-146. 73. Song Fanxu, Li Xiaojie, Cheng Chunfeng, et al. The Effect of Hydrotherapy on Gross Motor Function, Lower Limb Muscle Strength, and Muscle Tone in Children with Spastic Diplegic Cerebral Palsy. Chinese Journal of Integrated Traditional and Western Pediatrics, 2015, 7(4): 331-333. 74. Song Fanxu, Li Xiaojie, Cheng Chunfeng, et al. The Effect of Hydrotherapy on Gross Motor Function, Lower Limb Muscle Strength, and Muscle Tone in Children with Spastic Diplegic Cerebral Palsy. Chinese Journal of Integrated Traditional and Western Pediatrics, 2015, 7(4): 331-333. 75. Du Huiying, Chen Jing, Huang Qinru. Effect Analysis of Hydrotherapy Intervention on Gross Motor Function in Children with Central Coordination Disorders: A Study of 50 Cases. Shaanxi Medical Journal, 2012 (5): 627-628. 76. Wang Zhengxiang. Clinical Effect Analysis of Hydrotherapy Combined with Rehabilitation Nursing for Spastic Cerebral Palsy Children. Modern Medicine, 2014, 42(6): 661-663. 77. Zhang Rui. Application Observation of Hydrotherapy Combined with Sensory Integration Training in the Rehabilitation of Children with Cerebral Palsy. Chinese Journal of Recuperative Medicine, 2018, 27(10): 0-0. 78. Cui Yao, Xiao Dunwu, Ding Lin, Qiu Fubing, Cong Fang, Qiu Zhuoying. Systematic Review and Meta-analysis of the Effects of Aquatic Exercise Therapy on Motor Function and Activity Performance in Children and Adolescents with Cerebral Palsy. Chinese Journal of Rehabilitation Theory and Practice, 2021, 27(01): 79-92. 79. Cui Yao, Xiao Dunwu, Ding Lin, Qiu Fubing, Cong Fang, Qiu Zhuoying. Systematic Review and Meta-analysis of the Effects of Aquatic Exercise Therapy on Motor Function and Activity Performance in Children and Adolescents with Cerebral Palsy. Chinese Journal of Rehabilitation Theory and Practice, 2021, 27(01): 79-92. 80. Zhong Chen, Hu Shanshan, Zhang Hengshuo, et al. The Effect of Aquatic Therapy Combined with Routine Rehabilitation on Motor Function in Preschool Children with Spastic Cerebral Palsy. Chinese Journal of Children's Health Care, 2023, 31(5): 486. 81. Zhong Chen, Hu Shanshan, Zhang Hengshuo, et al. The Effect of Aquatic Therapy Combined with Routine Rehabilitation on Motor Function in Preschool Children with Spastic Cerebral Palsy. Chinese Journal of Children's Health Care, 2023, 31(5): 486. 82. Cui Yao, Xiao Dunwu, Ding Lin, et al. Systematic Review and Meta-analysis of the Effects of Aquatic Exercise Therapy on Motor Function and Activity Performance in Children and Adolescents with Cerebral Palsy. Chinese Journal of Rehabilitation Theory and Practice, 2021, 27(1): 79-92. 83. Cui Yao, Xiao Dunwu, Ding Lin, et al. Systematic Review and Meta-analysis of the Effects of Aquatic Exercise Therapy on Motor Function and Activity Performance in Children and Adolescents with Cerebral Palsy. Chinese Journal of Rehabilitation Theory and Practice, 2021, 27(1): 79-92. 84. Li Xiaoli, Tang Ting, Li Anfang. The Effect of Vortex Bubble Bath Hydrotherapy on Improving Motor Function in Children with Spastic Cerebral Palsy. International Medical and Health Journal, 2010, 16(3): 290-292. 85. Liu Zhenhuan, Dong Shangsheng. The Clinical Research on Early Intervention of Cerebral Palsy Using the Awakening Acupuncture Method to Prevent Disability. Proceedings of the 20th National Pediatric Chinese and Western Medicine Academic Conference, 2016. 86. Zhou Hongling, Zhou Tieying, Yu Yaping, et al. The Effect of Medicinal Hydrotherapy Combined with Functional Training on Improving Motor Function and Muscle Spasms in Children with Spastic Cerebral Palsy. Modern Chinese and Western Medicine Journal, 2004, 13(19): 2546-2546. 87. Zhou Hongling, Zhou Tieying, Yu Yaping, et al. The Effect of Medicinal Hydrotherapy Combined with Functional Training on Improving Motor Function and Muscle Spasms in Children with Spastic Cerebral Palsy. Modern Chinese and Western Medicine Journal, 2004, 13(19): 2546-2546. 88. Zhou Hongling, Zhou Tieying, Yu Yaping, et al. The Effect of Medicinal Hydrotherapy Combined with Functional Training on Improving Motor Function and Muscle Spasms in Children with Spastic Cerebral Palsy. Modern Chinese and Western Medicine Journal, 2004, 13(19): 2546-2546. 89. Tang Jiulai. Recent Advances in Rehabilitation Concepts and Techniques for Cerebral Palsy. Chinese Journal of Children's Health Care, 2017, 25(5): 433. 90. Li Chuyang, Liang Qi, Hou Xiaohui, et al. The Rehabilitation Effect of Swimming for School-Aged Children with Cerebral Palsy. Chinese Journal of Rehabilitation Theory and Practice, 2010, 16(7): 626-628. 91. Li Chuyang, Liang Qi, Hou Xiaohui, et al. The Rehabilitation Effect of Swimming for School-Aged Children with Cerebral Palsy. Chinese Journal of Rehabilitation Theory and Practice, 2010, 16(7): 626-628. 92. Qin Xiaojie, Peng Bo. The Application of Swimming Therapy in Rehabilitation Education for Children with Cerebral Palsy. Journal of Suihua University, 2020. 93. Fu Wenjie, Liu Zhenhuan, Jin Bingxu, et al. The Effect of Acupuncture Combined with Low-Frequency Electrical Stimulation on Lower Limb Muscle Tone in Children with Spastic Cerebral Palsy. Proceedings of the 20th National Pediatric Chinese and Western Medicine Academic Conference, 2016. 94. Fu Wenjie, Liu Zhenhuan, Jin Bingxu, et al. The Effect of Acupuncture Combined with Low-Frequency Electrical Stimulation on Lower Limb Muscle Tone in Children with Spastic Cerebral Palsy. Proceedings of the 20th National Pediatric Chinese and Western Medicine Academic Conference, 2016. 95. Zhang Jian, Yang Zheng, Liu Yumin. The Effect of Medicinal Hydrotherapy Combined with Functional Training on Motor Function in Children with Cerebral Palsy. Beijing Traditional Chinese Medicine Journal, 2007, 26(10): 664-664. 96. Dong Shangsheng. The Clinical Effect of Awakening Acupuncture Method Combined with NGF Acupoint Injection for Brain Injury Syndrome: Clinical Research. Guangzhou University of Chinese Medicine, 2015. 97. Dong Shangsheng. The Clinical Effect of Awakening Acupuncture Method Combined with NGF Acupoint Injection for Brain Injury Syndrome: Clinical Research. Guangzhou University of Chinese Medicine, 2015. |

| **Excluded articles (content not matching or inconsistent intervention/control measures based on abstract reading reviews, animal experiments, systematic reviews)(n =389)** | | | | |
| --- | --- | --- | --- | --- |
| **No.** | **Study Title (Translated)** | **First Author.** | **Language** | **Exclusion Reason** |
| 1 | Active exercise interventions improve gross motor function of ambulant/semi ambulant school-aged children with cerebral palsy: A systematic review | Clutterbuck, G. | English | systematic reviews |
| 2 | Activity in water based on the Halliwick method | [M. J. REID](https://onlinelibrary.wiley.com/authored-by/REID/M.+J.). | English | systematic reviews |
| 3 | Acupuncture for acute hordeolum | [Ke Cheng](https://www.cochranelibrary.com/cdsr/doi/10.1002/14651858.CD011075.pub2/information). | English | content not matching |
| 4 | Acute and Chronic Pain in Children and Adolescents With Cerebral Palsy: Prevalence, Interference, and Management | Katarina Ostojic. | English | content not matching |
| 5 | Adverse events in women and children who have received intrapartum antibiotic prophylaxis treatment: a systematic review | F Seedat. | English | systematic reviews |
| 6 | Aerobic exercise in patients with haemophilia: A systematic review on safety, feasibility and health effects | F Tomschi. | English | systematic reviews |
| 7 | Aerobic Exercises and Gross Motor Function in Spastic Cerebral Palsy | Riphah International University. | English | content not matching |
| 8 | Physical Activity and the Health of Wheelchair Users: A Systematic Review in Multiple Sclerosis, Cerebral Palsy, and Spinal Cord Injury | Shelley S. Selph. | English | systematic reviews |
| 9 | Airway clearance techniques compared to no airway clearance techniques for cystic fibrosis | [Louise Warnock](https://www.cochranelibrary.com/cdsr/doi/10.1002/14651858.CD001401.pub4/information#CD001401-cr-0004). | English | content not matching |
| 10 | Anthropometric characteristics, grip strength, and physical activity levels of children with physical disabilities: A case study | Micaela Dorfling. | English | Inconsistent intervention |
| 11 | Applications, indications, and effects of passive hydrotherapy WATSU (WaterShiatsu)-A systematic review and meta-analysis | [AM Schitter](https://scholar.google.com.hk/citations?user=zsEV2zcAAAAJ&hl=zh-CN&oi=sra). | English | systematic reviews |
| 12 | Approach to the rehabilitation of spasticity and neuromuscular disorders in children | [Ann Henderson Tilton](https://www.neurologic.theclinics.com/article/S0733-8619(03)00066-5/abstract). | English | inconsistent intervention |
| 13 | Aquatic Compared With Land-Based Exercises on Gross Motor Function of Children/Adolescents With Cerebral Palsy: A Systematic Review With Meta-Analysis | [Elton Pauluka](https://onlinelibrary.wiley.com/authored-by/Pauluka/Elton). | English | systematic reviews |
| 14 | Aquatic exercise for children with cerebral palsy | [Michelle Kelly.](https://onlinelibrary.wiley.com/authored-by/Kelly/Michelle) | English | systematic reviews |
| 15 | Aquatic exercises for children with muscular dystrophy and spinal muscular atrophy: A systematic review | Salem, Y. | English | systematic reviews |
| 16 | An Aquatic Physical Therapy Program at a Pediatric Rehabilitation Hospital: A Case Series | Fragala-Pinkham, Maria A. | English | content not matching |
| 17 | Aquatic Therapy for a Child with Type III Spinal Muscular Atrophy: A Case Report | [Yasser Salem](https://www.tandfonline.com/author/Salem,+Yasser). | English | content not matching |
| 18 | Aquatic therapy for spastic cerebral palsy: a scoping review | Aomeng Xiang. | English | systematic reviews |
| 19 | Aquatic Therapy in Children and Adolescents with Disabilities: A Scoping Review | Anna Ogonowska-Slodownik. | English | systematic reviews |
| 20 | Aquatic Therapy: Scientific Foundations and Clinical Rehabilitation Applications | Bruce E.Becker. | English | content not matching |
| 21 | Aquatots | Trevelyan J. | English | content not matching |
| 22 | Art therapy for people with dementia | [Sunita R Deshmukh](https://www.cochranelibrary.com/cdsr/doi/10.1002/14651858.CD011073.pub2/information#CD011073-cr-0002). | English | content not matching |
| 23 | Assessment and treatment of spasticity in children with cerebral palsy | Lidija Dimitrijević. | English | inconsistent intervention |
| 24 | Barriers and facilitators of sports in children with physical disabilities: a mixed-method study | [Eva A. Jaarsma](https://www.tandfonline.com/author/Jaarsma,+Eva+A). | English | content not matching |
| 25 | Beliefs about pain among professionals working with children with significant neurologic impairment | [Tim F Oberlander](https://www.cambridge.org/core/search?filters[authorTerms]=Tim F Oberlander&eventCode=SE-AU). | English | content not matching |
| 26 | Break dance significantly increases static balance in 9 years-old soccer players | Leonardo Ricotti. | English | inconsistent intervention |
| 27 | Calcium and vitamin D for increasing bone mineral density in premenopausal women | [Lucía Méndez-Sánchez](https://www.cochranelibrary.com/cdsr/doi/10.1002/14651858.CD012664.pub2/information#CD012664-cr-0004). | English | content not matching |
| 28 | Can using the Mollii Suit improve the function and muscle tone in young adults with cerebral palsy? | [A.Madondo](https://www.physiotherapyjournal.com/article/S0031-9406(25)00088-4/fulltext). | English | inconsistent intervention |
| 29 | Care Pathways in Rehabilitation for Children and Adolescents with Cerebral Palsy: Distinctiveness of the Adaptation to the Italian Context | Silvia Faccioli. | English | content not matching |
| 30 | Cell-Based Therapy Using Umbilical Cord Blood for Novel Indications in Regenerative Therapy and Immune Modulation: An Updated Systematic Scoping Review of the Literature | Mina Rizk . | English | systematic reviews |
| 31 | Cerebral Palsy in Pakistan: A Review of Study Approaches, Status of Research and Trends | Mahnoor Mughal. | English | systematic reviews |
| 32 | Childhood physical activity body contact risk: feasibility of a novel technique for objective measurements of impact speed, frequency, and intentionality | [P. E. Longmuir](https://onlinelibrary.wiley.com/authored-by/Longmuir/P.+E.). | English | content not matching |
| 33 | The Children's Dermatology Life Quality Index (CDLQI): initial validation and practical use | [M.S. Lewis-jones](https://onlinelibrary.wiley.com/authored-by/LEWIS‐JONES/M.S.). | English | content not matching |
| 34 | Chronic pain in children and young people with cerebral palsy: a narrative review of challenges, advances, and future directions | [Adrienne Harvey](https://link.springer.com/article/10.1186/s12916-024-03458-0#auth-Adrienne-Harvey-Aff1). | English | systematic reviews |
| 35 | Comment | MD Max. | English | systematic reviews |
| 36 | Complementary and alternative medicine use in adult cerebral palsy: Needs, barriers and impact on quality of life | Edward A Hurvitz. | English | content not matching |
| 37 | Complex regional pain syndrome in children: A collaborative rehabilitation approach in Tasmania and a survey of Australian clinical practice | Flett, P. | English | content not matching |
| 38 | Conservative, physical and surgical interventions for managing faecal incontinence and constipation in adults with central neurological diseases | [Claire L Todd](https://www.cochranelibrary.com/cdsr/doi/10.1002/14651858.CD002115.pub6/information#CD002115-cr-0004). | English | content not matching |
| 39 | [Chamber Divers: The Untold Story of the D-Day Scientists Who Changed Special Operations Forever](https://www.google.com/books?hl=zh-CN&lr=&id=daXLEAAAQBAJ&oi=fnd&pg=PA1&dq=Constipation+in+toddlers:+the+untold+story&ots=BcYlxF9N8n&sig=lNyeF5stPtv-EvLpiD-mwnfwPrg) | [R Lance](https://scholar.google.com.hk/citations?user=JJKxFDoAAAAJ&hl=zh-CN&oi=sra). | English | content not matching |
| 40 | Cerebral Palsy: Current Opinions on Definition, Epidemiology, Risk Factors, Classification and Treatment Options | [Małgorzata Sadowska](https://www.tandfonline.com/author/Sadowska,+Małgorzata). | English | systematic reviews |
| 41 | Desensitization of the gag reflex in an adult with cerebral palsy: a case report | [Julie A. Reid BAppSc GradDip](https://onlinelibrary.wiley.com/authored-by/Reid/Julie+A.). | English | content not matching |
| 42 | A systematic review of artificial intelligence for pediatric physiotherapy practice: Past, present, and future | Ravula Sahithya Ravali. | English | systematic reviews |
| 43 | The differential impact of several types of sports on pulmonary functions and respiratory muscle strength in boys aged 8-12 | [Özgür](https://journals.sagepub.com/doi/abs/10.3233/IES-192105#con1) Bostancı. | English | content not matching |
| 44 | Does therapy improve function in patients with hereditary spastic paraparesis? | H SHEAHAN. | English | content not matching |
| 45 | Effect of a program of short bouts of exercise on bone health in adolescents involved in different sports: the PRO-BONE study protocol | [D Vlachopoulos](https://scholar.google.com.hk/citations?user=uYWT1BcAAAAJ&hl=zh-CN&oi=sra). | English | content not matching |
| 46 | Effect of action observation therapy on motor function in children with cerebral palsy: a systematic review of randomized controlled trials with meta-analysis | [Naglaa](https://journals.sagepub.com/doi/full/10.1177/0269215520954345#con1) Abdelhaleem. | English | systematic reviews |
| 47 | Effect of an intensive and sequenced rehabilitation protocol on the functional outcome after single event multilevel surgery in cerebral palsy | [PS AJEESH](https://scholar.google.com.hk/citations?user=NsE8HxoAAAAJ&hl=zh-CN&oi=sra). | English | content not matching |
| 48 | The effect of aquatic high-intensity interval training on aerobic performance, strength and body composition in a non-athletic population: systematic review and meta-analysis | [Julie E](https://journals.sagepub.com/doi/full/10.1177/0269215518792039#con1) Depiazzi. | English | inconsistent intervention |
| 49 | Effect of aquatic therapy on head control in cerebral palsy children | AMA Mostafa. | English | inconsistent intervention |
| 50 | Effect of assisted walking-movement in patients with genetic and acquired neuromuscular disorders with the motorised Innowalk device: an international case study meta-analysis | [Caroline Schmidt-Lucke](https://peerj.com/articles/7098/author-1). | English | systematic reviews |
| 51 | Effect of botulinum toxin treatment in children with spastic cerebral palsy | H Colovic. | English | inconsistent intervention |
| 52 | Effect of case study versus video simulation on nursing students' satisfaction, self-confidence, and knowledge: a quasi-experimental study | Elizabeth K. Herron. | English | content not matching |
| 53 | The effect of combined training-constraint and bimanual use on hand function of children with cerebral palsy hemiparesis | M Holzer-Cohen. | English | inconsistent intervention |
| 54 | Effect of hip CPM on gross motor function and development of the hip joint: a single-center randomized controlled study on spastic cerebral palsy children with hip dysplasia | [Lulu Wang](https://loop.frontiersin.org/people/2065050). | English | inconsistent intervention |
| 55 | Effect of intensity and type of postsurgical rehabilitation on the outcome of patients with cerebral palsy undergoing SEMLS | Sharan, D. | English | content not matching |
| 56 | Effect of Mirror Therapy on Upper Limb Function in Children and Adolescents with Hemiplegic Cerebral Palsy: A Systematic Review and Meta-Analysis | [Zubina Khan](https://www.tandfonline.com/author/Khan,+Zubina). | English | systematic reviews |
| 57 | Effect of muscle strength training in children and adolescents with spastic cerebral palsy: A systematic review and meta-analysis | [J Merino-Andres](https://scholar.google.com.hk/citations?user=zPQbdLsAAAAJ&hl=zh-CN&oi=sra). | English | systematic reviews |
| 58 | Effect of oral cleansing in cerebral palsied children (application of a water jet device) | Y Isshiki. | English | inconsistent intervention |
| 59 | The effect of upper extremity myofascial surgery on muscle tone and functional outcome in persons with spastic cerebral palsy | [Deepak Sharan](https://www.bodyworkmovementtherapies.com/article/S1360-8592(18)30301-2/fulltext). | English | inconsistent intervention |
| 60 | The Effectiveness of Aquatic Therapy Based on the Halliwick Concept in Children with Cerebral Palsy: A Systematic Review | [Catalina Tapia](https://www.tandfonline.com/author/Tapia,+Catalina). | English | systematic reviews |
| 61 | Effectiveness of aquatic therapy interventions in the management of children with cerebral palsy: A systematic review | [Meysam Roostaei](https://www.tandfonline.com/author/Roostaei,+Meysam). | English | systematic reviews |
| 62 | The Effectiveness of Aquatic Therapy on Children with Cerebral Palsy on their Gross Motor Function: A Systematic Review | [A Orr](https://www.physiotherapyjournal.com/article/S0031-9406(24)00355-9/fulltext). | English | systematic reviews |
| 63 | Effectiveness of Mechanical Horse-Riding Simulator-Based Interventions in Patients with Cerebral Palsy-A Systematic Review and Meta-Analysis | Esteban Obrero-Gaitán. | English | systematic reviews |
| 64 | Effectiveness of Partial Body Weight-Supported Treadmill Training on Various Outcomes in Different Contexts among Children and Adolescents with Cerebral Palsy: A Systematic Review and Meta-Analysis | Abdulmajeed Alotaibi. | English | systematic reviews |
| 65 | Effectiveness of physiotherapy and conductive education interventions in children with cerebral palsy: A focused review | Anttila, Heidi. | English | systematic reviews |
| 66 | Effectiveness of surf therapy for children with disabilities | Emily D. Clapham. | English | inconsistent intervention |
| 67 | Effectiveness of treadmill training on gait function in children with cerebral palsy: meta-analysis | [Yong-Gu Han](https://pubmed.ncbi.nlm.nih.gov/?term=). | English | systematic reviews |
| 68 | Effectiveness of virtual reality in children and young adults with cerebral palsy: a systematic review of randomized controlled trial | Junior Vitorino Fandim. | English | inconsistent intervention |
| 69 | Effects of a movement and swimming program on vital capacity and water orientation skills of children with cerebral palsy | [Yeshayahu Hutzler](https://onlinelibrary.wiley.com/authored-by/Hutzler/Yeshayahu). | English | inconsistent intervention |
| 70 | Effects of an ergonomic program on the quality of life and work performance of university staff with physical disabilities: A clinical trial with three-month follow-up | Maria Ángela Ramalho-Pires de Almeida. | English | content not matching |
| 71 | Effects of Aquatic Intervention on Gross Motor Skills in Children with Cerebral Palsy: A Systematic Review | Meysam Roostaei. | English | systematic reviews |
| 72 | Effects of aquatic interventions in children with neuromotor impairments: A systematic review of the literature | Miriam Getz. | English | systematic reviews |
| 73 | The effects of different types of gait training on gait performance in children and young adults with cerebral palsy | Andreas Karamolegkos. | English | inconsistent intervention |
| 74 | Effects of education and support groups organized by IBCLCs in early postpartum on breastfeeding | Yi-Hua Lee. | English | content not matching |
| 75 | Effects of equine-assisted therapy on the functionality of individuals with disabilities: systematic review and meta-analysis | [Alessandra Prieto](https://www.tandfonline.com/author/Prieto,+Alessandra). | English | systematic reviews |
| 76 | Effects of hippotherapy and therapeutic horseback riding on postural control or balance in children with cerebral palsy: a meta-analysis | [Monika Zadnikar](https://onlinelibrary.wiley.com/authored-by/ZADNIKAR/MONIKA). | English | systematic reviews |
| 77 | Effects of hippotherapy on gross motor function in children and adolescents with cerebral palsy-a scoping review | [Panagiotis Plotas](https://link.springer.com/article/10.1186/s13052-024-01715-9#auth-Panagiotis-Plotas-Aff1-Aff2). | English | systematic reviews |
| 78 | Integrated Management in Cerebral Palsy: Musculoskeletal Surgery and Rehabilitation in Ambulatory Patients | [Erich Rutz](https://link.springer.com/chapter/10.1007/978-3-319-67858-0_22#auth-Erich-Rutz). | English | content not matching |
| 79 | Effects of Pediatric Rehabilitation on Children With Spastic Quadriplegia Primary to Seizure Disorder and Global Developmental Delay: A Case Report | Neha M. Chitlange. | English | content not matching |
| 80 | Effects of Physical Activity Intervention for Chinese People With Severe Mental Illness | [Daniel](https://journals.sagepub.com/doi/full/10.1177/1049731518804900#con1) Young. | English | content not matching |
| 81 | The effects of robotic gait neurorehabilitation and focal vibration combined treatment in adult cerebral palsy | Stela Rutović. | English | content not matching |
| 82 | The effects of Watsu therapy on autonomic cardiovascular modulation and flexibility of children with cerebral palsy | [E Tufekcioglu](https://scholar.google.com.hk/citations?user=hiB2PkUAAAAJ&hl=zh-CN&oi=sra). | English | content not matching |
| 83 | Effects That Different Types of Sports Have on the Hearts of Children and Adolescents and the Value of Two-Dimensional Strain-Strain-Rate Echocardiography | Fatih Köksal Binnetoğlu. | English | content not matching |
| 84 | Efficacy of acupotomy for cerebral palsy  A systematic review and meta-analysis | Kwon, Chan-Young. | English | systematic reviews |
| 85 | Efficacy of antiseptics in the prevention of post-operative infections of the proximal femur, hip and pelvis regions in orthopedic pediatric patients. Analysis of the first result | Kokavec M. | English | content not matching |
| 86 | Efficacy of Robot-Assisted Gait Therapy Compared to Conventional Therapy or Treadmill Training in Children with Cerebral Palsy: A Systematic Review with Meta-Analysis | Irene Cortés-Pérez. | English | systematic reviews |
| 87 | Efficacy of suit therapy on functioning in children and adolescents with cerebral palsy: a systematic review and meta-analysis | [Elisabete Martins](https://onlinelibrary.wiley.com/authored-by/Martins/Elisabete). | English | systematic reviews |
| 88 | [Efficiency of the pedagogical model of teaching the basic competitive swimming strokes to children with cerebral palsy](https://essuir.sumdu.edu.ua/handle/123456789/87706) | [Radziejowski, P.](https://essuir.sumdu.edu.ua/browse?type=author&value=Radziejowski, P.) | English | content not matching |
| 89 | Electromyographic analysis of quadriceps muscle among children with cerebral palsy underwater and on dry ground | T Trócoli. | English | content not matching |
| 90 | Energy Regulation in Young People | [Caroline J Dodd](https://pubmed.ncbi.nlm.nih.gov/?term=). | English | content not matching |
| 91 | Epidemiology and patient journey of Rett syndrome in the United States: a real-world evidence study | [Damian May](https://link.springer.com/article/10.1186/s12883-023-03181-y#auth-Damian-May-Aff1). | English | content not matching |
| 92 | [Evaluating intense rehabilitative therapies with and without acupuncture for children with cerebral palsy: a randomized controlled trial](https://www.sciencedirect.com/science/article/pii/S0003999311011105) | Duncan, B. | English | content not matching |
| 93 | Evidence based physical therapy in cerebral palsy: A systematic review of literature in an ICF framework | Inge Franki. | English | systematic reviews |
| 94 | Evidence-based management and motor rehabilitation of cerebral palsy children and adolescents: a systematic review | [Silvia Faccioli](https://loop.frontiersin.org/people/2156450). | English | systematic reviews |
| 95 | Exercise for pregnant women with pre‐existing diabetes for improving maternal and fetal outcomes | [Julie Brown](https://www.cochranelibrary.com/cdsr/doi/10.1002/14651858.CD012696.pub2/information#CD012696-cr-0002). | English | content not matching |
| 96 | Exercise interventions for cerebral palsy | Jennifer M Ryan. | English | systematic reviews |
| 97 | Exercise interventions improve gross motor function in school-aged children with cerebral palsy: A systematic review | Georgina Clutterbuck. | English | systematic reviews |
| 98 | Exercise intervention protocol in children and young adults with cerebral palsy: the effects of strength, flexibility and gait training on physical performance, neuromuscular mechanisms and cardiometabolic risk factors (EXECP) | [Pedro Valadão](https://link.springer.com/article/10.1186/s13102-021-00242-y#auth-Pedro-Valad_o-Aff1). | English | content not matching |
| 99 | Exercise therapy for chronic fatigue syndrome | [Lillebeth Larun](https://www.cochranelibrary.com/cdsr/doi/10.1002/14651858.CD003200.pub8/information#CD003200-cr-0004). | English | content not matching |
| 100 | Exercise therapy in juvenile idiopathic arthritis | [T Takken](https://scholar.google.com.hk/citations?user=HpPWrAQAAAAJ&hl=zh-CN&oi=sra). | English | systematic reviews |
| 101 | Exercise training for adults undergoing maintenance dialysis | [Amelie Bernier-Jean](https://www.cochranelibrary.com/cdsr/doi/10.1002/14651858.CD014653/information#CD014653-cr-0004) | English | content not matching |
| 102 | Exploring the use of Halliwick aquatic therapy in the rehabilitation of children with disabilities: A scoping review | [Stephanie Rohn](https://onlinelibrary.wiley.com/authored-by/Rohn/Stephanie). | English | systematic reviews |
| 103 | Facilitating physical activity in children with movement impairment | [G O'Malley](https://scholar.google.com.hk/citations?user=z6G86pgAAAAJ&hl=zh-CN&oi=sra). | English | content not matching |
| 104 | Factors that influence participation in physical activity for people with bipolar disorder: a synthesis of qualitative evidence | [Claire J McCartan](https://www.cochranelibrary.com/cdsr/doi/10.1002/14651858.CD013557.pub2/information#CD013557-cr-0004). | English | content not matching |
| 105 | Feasibility of Using the International Classification of Functioning, Disability and Health for Children and Youth (ICF-CY) as a Framework for Aquatic Activities: A Scoping Review | Merav Hadar-Frumer. | English | systematic reviews |
| 106 | Characteristics and Prognosis of Epilepsy in Children With Cerebral Palsy | Dimitrios I. Zafeiriou. | English | systematic reviews |
| 107 | First experience of using botulinum toxin in treatment of spastic cerebral palsy in the republic of moldova | T Cucu. | English | content not matching |
| 107 | Foot orthoses for treating paediatric flat feet | [Angela M Evans](https://www.cochranelibrary.com/cdsr/doi/10.1002/14651858.CD006311.pub3/information#CD006311-cr-0004). | English | content not matching |
| 109 | Gait analysis patterns and rehabilitative interventions to improve gait in persons with hereditary spastic paraplegia: a systematic review and meta-analysis | [Silvia Faccioli](https://loop.frontiersin.org/people/2156450)1. | English | systematic reviews |
| 110 | WITHDRAWN: Gait kinematic and electromyographic analysis on land and in water in healthy adolescents | Federica Camuncoli. | English | inconsistent intervention |
| 111 | Serious Game-based Intervention for Children with Developmental Disabilities | Kokol, Peter. | English | inconsistent intervention |
| 112 | HABIT-ILE: a randomised trial of hand arm bimanual intensive training including lower extremity training for children with bilateral cerebral palsy | Sakzewski, L. | English | inconsistent intervention |
| 113 | Habitual physical activity in Dutch children and adolescents with haemophilia | [W. G. Groen](https://onlinelibrary.wiley.com/authored-by/GROEN/W.+G.). | English | inconsistent intervention |
| 114 | [The Halliwick swimming method](https://journals.co.za/doi/pdf/10.10520/AJA03796175_1799) | AC Lochau. | English | systematic reviews |
| 115 | Health service use among adults with cerebral palsy: a mixed-methods systematic review | [Manjula Manikandan](https://onlinelibrary.wiley.com/authored-by/Manikandan/Manjula). | English | systematic reviews |
| 116 | Hippotherapy in adults with acquired brain injury: A systematic review | Jodie Marquez. | English | systematic reviews |
| 117 | Perceptions of Equine-Assisted Activities and Therapies by Parents and Children With Spinal Muscular Atrophy | Lemke, Danielle. | English | content not matching |
| 118 | Hyperbaric oxygen therapy for thermal burns | [Elmer Villanueva](https://www.cochranelibrary.com/cdsr/doi/10.1002/14651858.CD004727.pub2/information#CD004727-cr-0002). | English | content not matching |
| 119 | Hyperimmune immunoglobulin for people with COVID‐19 | [Catherine Kimber](https://pubmed.ncbi.nlm.nih.gov/?term=). | English | content not matching |
| 120 | Hypoxia-ischemia causes persistent movement deficits in a perinatal rabbit model of cerebral palsy: assessed by a new swim test | Matthew Derrick. | English | content not matching |
| 121 | Impact of a 12-month multifaceted neurological physiotherapy intervention on gross motor function in women with Rett syndrome | Alen Kapel. | English | content not matching |
| 122 | Impact of abobotulinumtoxinA injections on the need for orthopedic operations in children with spastic forms of cerebral palsy | [Zmanovskaya, V. A.](https://ui.adsabs.harvard.edu/search/q=author:) | English | content not matching |
| 123 | The Impact of Aquatic Exercise on Walking Ability, Quality of Life, and Enjoyment in Children with Cerebral Palsy: A Systematic Review | Miki Nakatani. | English | systematic reviews |
| 124 | Methods of Hydrokinesis Therapy for Children 3-5 Years with Cerebral Palsy of Spastic Form | Inna Taran. | English | content not matching |
| 125 | The importance of hydrokinetotherapy in rehabilitation activities for locomotor deficiencies in cerebral palsy | Ban R. | English | content not matching |
| 126 | Improvement of acupuncture on independent walking ability in children with cerebral paralysis | HM Li. | English | content not matching |
| 127 | Infirmary rehabilitation of school children with cerebral palsy | [A. Majnemer](https://onlinelibrary.wiley.com/authored-by/Majnemer/A.). | English | systematic reviews |
| 128 | Influence of a Balance Protocol on the gross motor function of children with Cerebral Palsy | Silvia Leticia Pavão. | English | content not matching |
| 129 | Influence of aquatic therapy in children and youth with cerebral palsy: A qualitative case study in a special education school | [C Barmatz](https://scholar.google.com.hk/citations?user=gTgcpPAAAAAJ&hl=zh-CN&oi=sra). | English | content not matching |
| 130 | The Role of Physiotherapy in the Management of Functional Neurological Disorder in Children and Adolescents | Yu-Na Kim. | English | content not matching |
| 131 | Interim PET‐results for prognosis in adults with Hodgkin lymphoma: a systematic review and meta‐analysis of prognostic factor studies | [Angela Aldin](https://www.cochranelibrary.com/cdsr/doi/10.1002/14651858.CD012643.pub3/information#CD012643-cr-0004). | English | systematic reviews |
| 132 | Interventions for preventing falls in Parkinson's disease | [Natalie E Allen](https://www.cochranelibrary.com/cdsr/doi/10.1002/14651858.CD011574.pub2/information#CD011574-cr-0004). | English | content not matching |
| 133 | Interventions for promoting physical activity in people with neuromuscular disease | [Katherine Jones](https://www.cochranelibrary.com/cdsr/doi/10.1002/14651858.CD013544.pub2/information#CD013544-cr-0004)a. | English | content not matching |
| 134 | nterventions implemented through sporting organisations for promoting healthy behaviour or improving health outcomes | [Rebecca K Hodder](https://www.cochranelibrary.com/cdsr/doi/10.1002/14651858.CD012170.pub2/information#CD012170-cr-0004). | English | content not matching |
| 135 | Interventions in outside‐school hours childcare settings for promoting physical activity amongst schoolchildren aged 4 to 12 years | [Rosa Virgara](https://www.cochranelibrary.com/cdsr/doi/10.1002/14651858.CD013380.pub2/information#CD013380-cr-0004). | English | content not matching |
| 136 | Interventions to improve gross motor performance in children with neurodevelopmental disorders: a meta- analysis | [Barbara R. Luca](https://link.springer.com/article/10.1186/s12887-016-0731-6#auth-Barbara_R_-Lucas-Aff1-Aff2-Aff3-Aff4)s. | English | systematic reviews |
| 137 | Metabolic Control in Patients With Phenylketonuria Pre- and Post-Sapropterin Loading Test | [Catarina](https://journals.sagepub.com/doi/full/10.1177/2326409818788898#con1) Sousa Barbosa. | English | content not matching |
| 138 | Leap Motion Controller Video Game-Based Therapy for Upper Extremity Motor Recovery in Patients with Central Nervous System Diseases. A Systematic Review with Meta-Analysis | Irene Cortés-Pérez . | English | systematic reviews |
| 139 | Limited Evidence of Functional Benefit After Upper Limb Botulinum Toxin Treatment in Children With Cerebral Palsy | Gresits, Orsolya Zsuzsanna. | English | systematic reviews |
| 140 | Macrolides versus placebo for chronic asthma | [Krishna Undela](https://www.cochranelibrary.com/cdsr/doi/10.1002/14651858.CD002997.pub5/information#CD002997-cr-0004). | English | content not matching |
| 141 | Malignant Otitis Externa in an Adult Diabetic Patient Caused by Escherichia coli: a Case Report | Franko Batinović. | English | content not matching |
| 142 | Management of infection after instrumented posterior spine fusion in pediatric scoliosis | Ho, Christine. | English | content not matching |
| 143 | Management of spinal infections in children with cerebral palsy | A.Sebaaly. | English | content not matching |
| 144 | A meta‐ethnography of how children and young people with chronic non‐cancer pain and their families experience and understand their condition, pain services, and treatments | [Emma France](https://www.cochranelibrary.com/cdsr/doi/10.1002/14651858.CD014873.pub2/information#CD014873-cr-0004). | English | content not matching |
| 145 | Micronutrient supplementation in adults with HIV infection | [Marianne E Visser](https://www.cochranelibrary.com/cdsr/doi/10.1002/14651858.CD003650.pub4/information#CD003650-cr-0002). | English | content not matching |
| 146 | Moving together is better: a systematic review with meta-analysis of sports-focused interventions aiming to improve physical activity participation in children and adolescents with cerebral palsy | Ricardo Rodrigues de Sousa Junior. | English | systematic reviews |
| 147 | Muscle Strengthening in Children and Adolescents With Spastic Cerebral Palsy: Considerations for Future Resistance Training Protocols | Olaf Verschuren. | English | content not matching |
| 148 | Myofascial structural integration therapy on gross motor function and gait of young children with spastic cerebral palsy: a randomized controlled trial | [Elizabeth C. Loi](https://loop.frontiersin.org/people/256243). | English | inconsistent intervention |
| 149 | Nationwide Survey Reveals High Prevalence of Non-Swimmers among Children with Congenital Heart Defects | Christian Apitz. | English | content not matching |
| 150 | Natural History of Chronic Pain and Pain Treatment in Adults with Cerebral Palsy | Jensen, Mark. | English | content not matching |
| 151 | Neurodegeneration with brain iron accumulation: a differential diagnosis of cerebral palsy | [Alana Ferreira de Andrade](https://link.springer.com/article/10.1186/s41983-023-00639-1#auth-Alana-Ferreira_de_Andrade-Aff1). | English | content not matching |
| 152 | Neurodevelopmental Treatment Approach for Teaching Swimming to Cerebral Palsied Children | [Susan R. Harris](javascript:;). | English | systematic reviews |
| 153 | Early mobilisation by locomotion therapy following minimally invasive multi-level surgery for children and young adults with cerebral palsy. | [D SHARAN](https://scholar.google.com.hk/citations?user=KLqdqvYAAAAJ&hl=zh-CN&oi=sra). | English | content not matching |
| 154 | Neurotic, neuromuscular and autonomic nervous form of magnesium imbalance | Durlach J. | English | content not matching |
| 155 | Next‐generation sequencing for guiding matched targeted therapies in people with relapsed or metastatic cancer | [Farasat Kazmi](https://pubmed.ncbi.nlm.nih.gov/?term=). | English | content not matching |
| 156 | Nintendo Wii Balance Board therapy for postural control in children with cerebral palsy: a systematic review and meta-analysis | [Desirée Montoro-Cárdenas](https://onlinelibrary.wiley.com/authored-by/Montoro‐Cárdenas/Desirée). | English | systematic reviews |
| 157 | No Evidence to Support Aquatic Therapy for Children With Cerebral Palsy, But Do Their Experiences Matter More? | Williamson, Eric. | English | systematic reviews |
| 158 | Non‐biologic, steroid‐sparing therapies for non‐infectious intermediate, posterior, and panuveitis in adults | Rebecca G Edwards Mayhew. | English | content not matching |
| 159 | Non‐nutritive sweeteners for diabetes mellitus | [Szimonetta Lohner](https://www.cochranelibrary.com/cdsr/doi/10.1002/14651858.CD012885.pub2/information#CD012885-cr-0004). | English | content not matching |
| 160 | Omega 3 fatty acids (fish oil) for maintenance of remission in Crohn's disease | [Dan Turner](https://www.cochranelibrary.com/cdsr/doi/10.1002/14651858.CD006320.pub3/information#CD006320-cr-0002). | English | content not matching |
| 161 | Oral myiasis: A case report | AP Bhatt. | English | content not matching |
| 162 | Outcomes of Bone Marrow Mononuclear Cell Transplantation for Neurological Sequelae Due to Intracranial Hemorrhage Incidence in the Neonatal Period: Report of Four Cases | Nguyen Thanh Liem. | English | content not matching |
| 163 | Oxygen uptake in spastic cerebral palsy during normal gait and hydrotherapy gait | [N Suzuki](https://scholar.google.com.hk/citations?user=AIyEK4MAAAAJ&hl=zh-CN&oi=sra). | English | inconsistent intervention |
| 164 | Pain with cerebral palsy: Health professionals' assessment and treatment practices and the relationship to beliefs survey | P Crosato. | English | content not matching |
| 165 | Parental autoimmune and autoin flam matory disorders as multiple risk factors for common neurodevelopmental disorders in offspring: a systematic review and meta-analysis | [Pierre Ellul](https://www.nature.com/articles/s41398-022-01843-y#auth-Pierre-Ellul-Aff1-Aff2). | English | systematic reviews |
| 166 | Performance of Positron Emission Tomography at Diagnosis and Response Assessment in Low and Intermediate-Risk Nodular Lymphocyte Predominant Hodgkin Lymphoma | Lianna J.Marks. | English | content not matching |
| 167 | Personalised care planning for adults with chronic or long‐term health conditions | [Angela Coulter](https://www.cochranelibrary.com/cdsr/doi/10.1002/14651858.CD010523.pub2/information#CD010523-cr-0002). | English | content not matching |
| 168 | Pharmacological treatment of gastro‐oesophageal reflux in children | [Mark P Tighe](https://www.cochranelibrary.com/cdsr/doi/10.1002/14651858.CD008550.pub3/information#CD008550-cr-0004). | English | content not matching |
| 169 | Physical activity and exercise training in cystic fibrosis | [Thomas Radtke](https://www.cochranelibrary.com/cdsr/doi/10.1002/14651858.CD002768.pub5/information#CD002768-cr-0004). | English | content not matching |
| 170 | Physical activity and rural middle school adolescents | [Michael P. Savage](https://link.springer.com/article/10.1023/A:1021619930697#auth-Michael_P_-Savage). | English | content not matching |
| 171 | Physical Activity and the Health of Wheelchair Users: A Systematic Review in Multiple Sclerosis, Cerebral Palsy, and Spinal Cord Injury | Shelley S. Selph. | English | systematic reviews |
| 172 | Physical activity for treatment of irritable bowel syndrome | [David Nunan](https://www.cochranelibrary.com/cdsr/doi/10.1002/14651858.CD011497.pub2/information#CD011497-cr-0004). | English | content not matching |
| 173 | Physical activity, hydration and health | [Marcos, Ascensión](https://digibug.ugr.es/browse?authority=dd7170d7-1718-4d09-9ded-195d3031f602&type=author). | English | content not matching |
| 174 | Physical exercise for people with Parkinson’s disease: a systematic review and network meta‐analysis | [Moritz Ernst](https://www.cochranelibrary.com/cdsr/doi/10.1002/14651858.CD013856.pub3/information#CD013856-cr-0004). | English | systematic reviews |
| 175 | Physical therapy in children with cerebral palsy in Brazil: a scoping review | [Michelle A S Furtado](https://onlinelibrary.wiley.com/authored-by/Furtado/Michelle+A+S). | English | content not matching |
| 176 | Physical therapy interventions to improve sitting ability in children with or at-risk for cerebral palsy: a systematic review and meta-analysis | [Ketaki Inamdar](https://onlinelibrary.wiley.com/authored-by/Inamdar/Ketaki). | English | systematic reviews |
| 177 | A guide to physiotherapy in cerebral palsy | Christine E. Barber. | English | systematic reviews |
| 178 | Knowledge and perception of physiotherapy among healthcare science students at the Maldives National University | [Isna Abdul Qayyoom](https://link.springer.com/article/10.1186/s43161-024-00253-z#auth-Isna_Abdul-Qayyoom-Aff1). | English | content not matching |
| 179 | Physiotherapy of cerebral movement disorders in childhood | [H Aurich](https://pubmed.ncbi.nlm.nih.gov/?size=100&term=Aurich+H&cauthor_id=366235). | English | systematic reviews |
| 180 | Position Stand on Androgen and Human Growth Hormone Use | Hoffman, Jay R. | English | content not matching |
| 181 | Efeito postural agudo da fisioterapia aquática na encefalopatia crônica não progressiva da infância | Bruna Yamaguchi. | English | inconsistent intervention |
| 182 | Postural balance control interventions in autism spectrum disorder (ASD): A systematic review | Surabhi Date . | English | systematic reviews |
| 183 | Physical activity for treatment of irritable bowel syndrome | [David Nunan](https://www.cochranelibrary.com/cdsr/doi/10.1002/14651858.CD011497.pub2/information#CD011497-cr-0004). | English | content not matching |
| 184 | Physical activity, hydration and health | Marcos, Ascensión. | English | content not matching |
| 185 | Physical and Psychosocial Benefits of Sports Participation Among Children and Adolescents with Chronic Diseases: A Systematic Review | [Borja Sañudo](https://link.springer.com/article/10.1186/s40798-024-00722-8#auth-Borja-Sa_udo-Aff1). | English | systematic reviews |
| 186 | Physical exercise for people with Parkinson’s disease: a systematic review and network meta‐analysis | Moritz Ernst. | English | systematic reviews |
| 187 | Physical therapy in children with cerebral palsy in Brazil: a scoping review | MAS Furtado. | English | systematic reviews |
| 188 | Physical therapy interventions to improve sitting ability in children with or at-risk for cerebral palsy: a systematic review and meta-analysis | [Ketaki Inamdar](https://onlinelibrary.wiley.com/authored-by/Inamdar/Ketaki). | English | systematic reviews |
| 189 | Efficacy of intensive versus nonintensive physiotherapy in children with cerebral palsy: a meta-analysis | Arpino, Carla. | English | systematic reviews |
| 190 | Exploring Access to Health-Related Rehabilitation Services for Persons with Disabilities in the Maldives | T O'Fallon. | English | content not matching |
| 191 | Physiotherapy of cerebral movement disorders in childhood | H Aurich. | English | content not matching |
| 192 | Position Stand on Androgen and Human Growth Hormone Use | Hoffman, Jay R. | English | content not matching |
| 193 | Effect of aquatic physical therapy on pain and state of sleep and wakefulness among stable preterm newborns in neonatal intensive care units | C Vignochi. | English | content not matching |
| 194 | Postural balance control interventions in autism spectrum disorder (ASD): A systematic review | Surabhi Date. | English | systematic reviews |
| 195 | Omega-3 fatty acids: Mechanisms of benefit and therapeutic effects in pediatric and adult NAFLD | [Valerio Nobili](https://www.tandfonline.com/author/Nobili,+Valerio). | English | content not matching |
| 196 | The power of Para sport: the effect of performance-focused swimming training on motor function in adolescents with cerebral palsy and high support needs (GMFCS IV) - a single-case experimental design with 30-month follow-up | Iain Mayank Dutia[1](https://bjsm.bmj.com/content/58/14/777.abstract#aff-1). | English | content not matching |
| 197 | Pre-Participation Medical Evaluation for Adventure and Wilderness Watersports | Andrew T. Nathanson. | English | inconsistent intervention |
| 198 | Sensorimotor interventions and assessments for the hand and wrist: A scoping review | Kristin Valdes. | English | systematic reviews |
| 199 | Study on the Role of Physical Education in Educating Psycho Motility for Children with Special Needs | Elena Moldovan. | English | content not matching |
| 200 | Pulmonary rehabilitation versus usual care for adults with asthma | [Christian R Osadnik](https://www.cochranelibrary.com/cdsr/doi/10.1002/14651858.CD013485.pub2/information#CD013485-cr-0004). | English | content not matching |
| 201 | Randomized controlled trial of traditional Chinese medicine (acupuncture and Tuina) in cerebral palsy: Part 1 - Any increase in seizure in integrated acupuncture and rehabilitation group versus rehabilitation group? | [YunWu](https://www.liebertpub.com/doi/abs/10.1089/acm.2007.0756#con1). | English | content not matching |
| 202 | Recent advancements in interventions for cerebral palsy – A review | Priya Sharma. | English | systematic reviews |
| 203 | Recruitment of infants with sickle cell anemia to a Phase III trial: data from the BABY HUG study | Lynn Wynn. | English | content not matching |
| 204 | Rehabilitation effect of aquatic exercise on motor function in children with cerebral palsy: Systematic review of randomized controlled trials | [Fangqin](https://journals.sagepub.com/doi/abs/10.1177/10538127251313951#con1) Li. | English | systematic reviews |
| 205 | Rehabilitation is growing in europe: A particular role for ESPRM | Giustini, Alessandro. | English | content not matching |
| 206 | Rett syndrome. A review with emphasis on clinical characteristics and intervention | [Meir Lotan](https://onlinelibrary.wiley.com/authored-by/Lotan/Meir), | English | systematic reviews |
| 207 | Reversal of an unintentional spinal anesthetic by cerebrospinal lavage | Tsui, Ban C. H. | English | content not matching |
| 208 | Role-play versus lecture methods in community health volunteers | Fatemeh Vizeshfar. | English | content not matching |
| 209 | Exercise Training Program in Children and Adolescents With Cerebral PalsyA Randomized Controlled Trial | [Olaf Verschuren](https://jamanetwork.com/searchresults?author=Olaf+Verschuren&q=Olaf+Verschuren). | English | content not matching |
| 210 | Safety and efficacy of proton pump inhibitors in preterm infants with gastroesophageal reflux disease | [Esther King](https://pubmed.ncbi.nlm.nih.gov/?term=). | English | content not matching |
| 211 | Saline irrigation for allergic rhinitis | [Karen Head](https://www.cochranelibrary.com/cdsr/doi/10.1002/14651858.CD012597.pub2/information#CD012597-cr-0002). | English | content not matching |
| 212 | Sanatorium-health resort treatment of children with cerebral palsy in Evpatoria | Koreniuk, S.V. | English | content not matching |
| 213 | Short- to Long-Term Effects of Virtual Reality on Motor Skill Learning in Children With Cerebral Palsy: Systematic Review and Meta-Analysis | [Seyma Kilcioglu](https://games.jmir.org/search?term=Seyma Kilcioglu&type=author&precise=true&authorlink=true). | English | systematic reviews |
| 214 | Short-Term Effects of Vestibular Training on Gross Motor Function in Children and Youth with Cerebral Palsy: A Systematic Review and Meta-Analysis of Randomized Clinical Trials | María Coello-Villalón. | English | systematic reviews |
| 215 | Social participation: the perspectives of adolescents with cerebral palsy and their mothers | [Priscila Bianchi Lopes](https://www.tandfonline.com/author/Lopes,+Priscila+Bianchi). | English | content not matching |
| 216 | SOFTWARE AND HARDWARE APPLICATIONS TO THE KINESITHERAPY OF CHILDREN WITH CEREBRAL PALSY. | Necula, Dana. | English | inconsistent intervention |
| 217 | Spatiotemporal analysis and clinical findings on gait. Comparison of two treatment modalities in children with spastic hemiplegia cerebral palsy. Preliminary report | Arellano-Martínez IT. | English | inconsistent intervention |
| 218 | SPLASH study: Exploring caregiver perspectives of adults with severe or profound and multiple learning disabilities accessing sedentary hydrotherapy | [C. Tbaily](https://www.physiotherapyjournal.com/article/S0031-9406(21)00608-8/fulltext). | English | content not matching |
| 219 | Sport activity of children and adolescents with hemophilia | Iwona SZYMKUĆ-BUKOWSKA. | English | content not matching |
| 220 | Sports participation of children with Down syndrome | Winell J. | English | content not matching |
| 221 | SPORTS STARS study protocol: a randomised, controlled trial of the effectiveness of a physiotherapist-led modified sport intervention for ambulant school-aged children with cerebral palsy | [Georgina L.Clutterbuck](https://link.springer.com/article/10.1186/s12887-018-1190-z#auth-Georgina_L_-Clutterbuck-Aff1-Aff2) | English | content not matching |
| 222 | Standardized Outcomes Measures in Physical Therapy Practice for Treatment and Rehabilitation of Cerebral PALSY: A Systematic Review | Maria Dolores Apolo-Arenas. | English | systematic reviews |
| 223 | Strength training and aerobic exercise training for muscle disease | [Nicoline BM Voet](https://www.cochranelibrary.com/cdsr/doi/10.1002/14651858.CD003907.pub5/information#CD003907-cr-0002). | English | content not matching |
| 224 | Summative evaluation of a pilot aquatic exercise program for children with disabilities | Maria Fragala-Pinkham. | English | inconsistent intervention |
| 225 | A Qualitative Investigation of Swimming Experiences of Children With Autism Spectrum Disorders and Their Families | [Lisa](https://journals.sagepub.com/doi/full/10.1177/1179556519872214#con1) Mische Lawson. | English | content not matching |
| 226 | A systematic review of interventions for children with cerebral palsy: State of the evidence | [Iona Novak](https://onlinelibrary.wiley.com/authored-by/Novak/Iona). | English | systematic reviews |
| 227 | A systematic review of the effectiveness of aerobic exercise interventions for children with cerebral palsy: an AACPDM evidence report | [Anna Rogers](https://onlinelibrary.wiley.com/authored-by/Rogers/Anna). | English | systematic reviews |
| 228 | Time cost associated with sports participation for athletes with high support needs: a time-motion analysis of tasks required for para swimming | Iain Dutia. | English | content not matching |
| 229 | Traditional Chinese medicine for treatment of cerebral palsy in children: A systematic review of randomized clinical trials | [Yuqing](https://www.liebertpub.com/doi/abs/10.1089/acm.2009.0609#con1) Zhang. | English | systematic reviews |
| 230 | Treatments for women with gestational diabetes mellitus: an overview of Cochrane systematic reviews | [Ruth Martis](https://www.cochranelibrary.com/cdsr/doi/10.1002/14651858.CD012327.pub2/information#CD012327-cr-0002). | English | systematic reviews |
| 231 | The underwater treadmill as a physical therapy intervention for children with cerebral palsy | [Michael Hill](https://faseb.onlinelibrary.wiley.com/authored-by/Hill/Michael). | English | systematic reviews |
| 232 | Ventilation tubes (grommets) for otitis media with effusion (OME) in children | [Samuel MacKeith](https://www.cochranelibrary.com/cdsr/doi/10.1002/14651858.CD015215.pub2/information#CD015215-cr-0004). | English | content not matching |
| 233 | Virtual reality distraction for acute pain in children | Veronica Lambert. | English | content not matching |
| 234 | Vitamin D supplementation for the treatment of COVID‐19: a living systematic review | [Julia Kristin Stroehlein](https://www.cochranelibrary.com/cdsr/doi/10.1002/14651858.CD015043/information#CD015043-cr-0004). | English | systematic reviews |
| 235 | Effects of Exercise Training on Performance and Function in Individuals with Cerebral Palsy: A Critical Review | S. Ferreira. | English | systematic reviews |
| 236 | Effect of Aerobic Capacity on Health-Related Quality of Life in Adults with Cerebral Palsy | Y. You. | English | systematic reviews |
| 237 | Aerobic Capacity in Children and Adolescents with Cerebral Palsy | O. Verschuren. | English | content not matching |
| 238 | Challenges of Spa Tourism in the Republic of Serbia for the Rehabilitation of Children with Motor Skills Problems | A. Anufrijev. | English | content not matching |
| 239 | Radial Extracorporeal Shock Wave Therapy (rESWT) in the Treatment of Spasticity in Cerebral Palsy: A Randomized, Placebo-Controlled Clinical Trial | X. Vidal. | English | content not matching |
| 240 | Water-Based Interventions for People With Neurological Disability, Autism, and Intellectual Disability: A Scoping Review | Karlee Naumann. | English | systematic reviews |
| 241 | The therapeutic effects of physical treatment for patients with hereditary spastic paraplegia: a narrative review | [Armando Di Ludovico](https://loop.frontiersin.org/people/1919803)1. | English | systematic reviews |
| 242 | Treatment of Cerebral Palsy in the Severely Subnormal | E.D.R. Campbell. | English | content not matching |
| 243 | Principles for the Use of Rehabilitation Aids in Children Aged 0–3 Years with Cerebral Palsy | Zhifang Cao | Chinese | content not matching |
| 244 | Early Screening and Intervention for High-Risk Infants with Cerebral Palsy Aged 0–6 Months | Yuhui You | Chinese | content not matching |
| 245 | Evaluation of Short- and Long-Term Motor and Cognitive Development after Hypoxic-Ischemic Brain Injury in 3-Day-Old Immature Rats | Lan Hu | Chinese | content not matching |
| 246 | Clinical Analysis of 208 Cases of Children with Cerebral Palsy | Ying Li | Chinese | content not matching |
| 247 | Sinicization and Reliability Study of the Alyn Water Adaptation Scale 1 | Long Jin, | Chinese | content not matching |
| 248 | Effects of Botulinum Toxin Type A Injection Combined with Functional Training on Motor Ability in Children with Spastic Cerebral Palsy | Xiaobo Jiao | Chinese | content not matching |
| 249 | Observation on the Rehabilitation Effect of Halliwick Technique Combined with Aquatic Play Training in Children with Motor Developmental Delay | Chaochao Sun | Chinese | content not matching |
| 250 | Observation on the Therapeutic Effects of Three Intervention Routes of HUC-MSCs on Hypoxic-Ischemic Brain Injury in Infant Rats | Linyan Zhou | Chinese | content not matching |
| 251 | Study on the Neuroprotective Effects of p38MAPK Inhibitor after Reoxygenation following Hypoxia in Zebrafish Larvae | Jun Chen | Chinese | content not matching |
| 252 | Resource Linking and Integration in Medical Social Work from an Ethnographic Perspective | Chenxi Li | Chinese | content not matching |
| 253 | Therapeutic Effect of Moxibustion with Buzhong Yiqi Decoction Containing Different Doses of Astragalus on Knee Hyperextension in Children with Spastic Cerebral Palsy | Ling Chen | Chinese | content not matching |
| 254 | Observation on the Therapeutic Effect of Herbal Steam Bath in Children with Spastic Cerebral Palsy | Zhongmin You | Chinese | content not matching |
| 255 | Evaluation of the Effect of Herbal Bathing on Brachial Artery Blood Flow Using Color Doppler Ultrasound | Xiaoli Hu, | Chinese | content not matching |
| 256 | Physical Activity and Its Health Effects in Children and Adolescents with Disabilities: A Systematic Review of Systematic Reviews | Tongnian Yang, | Chinese | systematic reviews |
| 257 | Health and Functional Benefits of Aquatic Rehabilitation in Children with Disabilities: A Systematic Review of Systematic Reviews Based on ICD-11 and ICF Frameworks | Ting Liao, | Chinese | systematic reviews |
| 258 | Effects of Congnao Tongluo Method Combined with Buzhong Yiqi Decoction Bath on Children with Spastic Cerebral Palsy | Ju Dang, | Chinese | content not matching |
| 259 | Effects of Congnao Tongluo Acupuncture Combined with Peony and Licorice Decoction Fumigation Bath on Cerebral Blood Flow and Motor Ability in Children with Spastic Cerebral Palsy | Biyu Lin | Chinese | content not matching |
| 260 | Study on the Synthesis of Cannabidiol and Its Antidepressant Effects | Melkamu Alemu Abame | Chinese | content not matching |
| 261 | Study on Physical Growth and Neurobehavioral Development in Low Birth Weight Infants | Yuanyuan Su | Chinese | content not matching |
| 262 | Observation on the Therapeutic Effect of Acupressure Therapy on Spastic Muscles in Children with Early Cerebral Palsy | Zhen Zeng | Chinese | content not matching |
| 263 | Effects of Electroacupuncture and Massage Combined with Rehabilitation Training on Spasticity and Motor Ability in Children with Spastic Cerebral Palsy | Qiong Wang, | Chinese | content not matching |
| 264 | Effects of Electroacupuncture at Huatuojiaji Points Combined with Acupoint Injection on Cerebral Blood Circulation in Children with Cerebral Palsy | Ruili | Chinese | content not matching |
| 265 | Evaluation of the Effect of Herbal Bathing on Brachial Artery Blood Flow Using Color Doppler Ultrasound | Xiaoli Hu | Chinese | content not matching |
| 266 | Effects of Rehabilitation Models on Recovery Outcomes in Children with Cerebral Palsy | Lihua Xie | Chinese | content not matching |
| 267 | A Case Report of Hypoxic Brain Injury in a Child after Drowning | Sha Dong | Chinese | content not matching |
| 268 | Rehabilitation Treatment of Children with Cerebral Palsy | Xiujie Xie | Chinese | content not matching |
| 269 | Study on the Mechanisms of Motor Control Deficits in Children with Developmental Coordination Disorder | Jing Hua | Chinese | content not matching |
| 270 | Intervention Effects of Enriched Environmental Stimulation and Hydrotherapy on Hypoxic-Ischemic Brain Injury in Neonatal Rats | Xiaorong Liu | Chinese | content not matching |
| 271 | Effects of Comprehensive Hyperbaric Oxygen Therapy on Cognitive, Speech, and Motor Abilities in Children with Early Cerebral Palsy | Ying Xian, | Chinese | content not matching |
| 272 | Analysis of the Intervention Effect of Personalized Rehabilitation Nursing on Life and Motor Abilities in Children with Cerebral Palsy | Lingjun Guo | Chinese | content not matching |
| 273 | Effects of Personalized Aquatic Play Intervention on Children with Global Developmental Delay | Tiantian Lu | Chinese | inconsistent intervention |
| 274 | Study on the Classical Wnt Signaling Pathway and Oxidative Stress Changes in an Autism Model of Rats | Yinghua Zhang | Chinese | content not matching |
| 275 | Study on the Effects of Core Stability Training Combined with Meridian-Based Massage on Muscle Tone and Motor Ability in Children with Spastic Cerebral Palsy | Ping Li, Jing | Chinese | inconsistent intervention |
| 276 | Meta-analysis on the Effect of Core Stability Training on Motor Ability in Children with Spastic Cerebral Palsy | Jinghui Deng | Chinese | systematic reviews |
| 277 | Effects of Robot-Assisted Therapy Combined with Electronic Biofeedback on Rehabilitation in Children with Brain Injury | Xinxia Lou | Chinese | content not matching |
| 278 | Motor Function Assessment and Hydrotherapy Program for Children with Cerebral Palsy Based on the ICF-CY Framework | Guoxiang Wang | Chinese | systematic reviews |
| 279 | Clinical Study of Laser Acupuncture and Traditional Acupuncture in the Treatment of Children with Cerebral Palsy | Yonglin Lu | Chinese | inconsistent intervention |
| 280 | Effects of Jian’nao Yizhi Powder Combined with Acupuncture on Motor, Language, and Daily Living Abilities in Children with Cerebral Palsy | Yuanjun Lou | Chinese | content not matching |
| 281 | Spreading Love into Every Child's Heart: A Chronicle of the Establishment of Shenyang Special Education School for Orphans and Disabled Children | Yanping Li | Chinese | content not matching |
| 282 | Clinical and Experimental Study of Jin San Zhen Acupuncture Therapy in the Treatment of Children with Cerebral Palsy | Ruitao She | Chinese | content not matching |
| 283 | Research Progress on the Correction of Abnormal Gait in Children with Spastic Cerebral Palsy | Kai Lu | Chinese | content not matching |
| 284 | Effects of Rehabilitation Training Combined with Botulinum Toxin Type A on Spasticity and Gross Motor Function in Children with Spastic Cerebral Palsy | Guixian Wang | Chinese | content not matching |
| 285 | Clinical Research Progress on Early Intervention for Children with Brain Injury Syndrome | Qiaoling Xie | Chinese | content not matching |
| 286 | Early Rehabilitation Research and Progress on Cerebral Palsy | Yongfeng Hong | Chinese | content not matching |
| 287 | Respiratory Function Characteristics in Children with Cerebral Palsy and the Role of Hydrotherapy | Wenzhe Hao | Chinese | inconsistent intervention |
| 288 | Research Progress on the Mechanism and Treatment Strategies of Equinovarus Deformity in Children with Cerebral Palsy | Yiwen | Chinese | content not matching |
| 289 | Design and Construction of Rehabilitation Training Rooms for Children with Cerebral Palsy | Yiru Lu | Chinese | content not matching |
| 290 | Rehabilitation Treatment Effects for High-Risk Children with Cerebral Palsy | Jiujun Qiu | Chinese | content not matching |
| 291 | Effects of Pentoxifylline Pretreatment on DA Neuron Function in the Midbrain of Epileptic Rats and the Role of Nrf2-ARE Pathway | Yunxiao Kang | Chinese | content not matching |
| 292 | Strengthening the Management of High-Risk Infants to Promote Growth, Development, and Intelligence | Zhaoling Meng | Chinese | content not matching |
| 293 | Effects of Home-Based Rehabilitation on Motor Ability in Children with Cerebral Palsy | Maoqun Ran | Chinese | content not matching |
| 294 | Observation on the Effects of Home Rehabilitation Training on Gross Motor Function in Children with Spastic Diplegic Cerebral Palsy | Ping Yao | Chinese | content not matching |
| 295 | Effects of Home Training and Parental Cooperation on Early Cognitive Development in Children with Developmental Delay | Shuigui Yin | Chinese | content not matching |
| 296 | Observation on the Effect of a Home-Based Training Program on Fine Motor Skills in Children with Cerebral Palsy | Niannian Lin | Chinese | content not matching |
| 297 | Screening and Intervention for High-Risk Children with Cerebral Palsy | Yanqiong Dai | Chinese | content not matching |
| 298 | "Three-Level" Standardized Program for Cerebral Palsy Rehabilitation — Insights from the Rehabilitation Practice of Children with Cerebral Palsy in Gansu Pingliang Children’s Welfare Institute | Xialin Yang, | Chinese | content not matching |
| 299 | Discussion on the Rehabilitation of Children with Cerebral Palsy through Integrated Chinese and Western Medicine | Zhenhuan Liu | Chinese | content not matching |
| 300 | The Latest Advances in the Concepts and Techniques of Cerebral Palsy Rehabilitation | Jiu-lai Tang | Chinese | content not matching |
| 301 | Application of Bubble Bath Hydrotherapy in Pediatric Health Clinics | Ningning Peng | Chinese | systematic reviews |
| 302 | The Effects of Stretching and Weight-Bearing Rehabilitation Training on Motor Function and Intellectual Development in Children with Cerebral Palsy | Jing Zhou | Chinese | content not matching |
| 303 | The Effect of Whole-Body Vibration Training Combined with Abdominal Binder on Motor Function in Children with Spastic Cerebral Palsy | Xin Li | Chinese | content not matching |
| 304 | Application Experience of Sensory Integration Training Combined with Neurodevelopmental Therapy in Rehabilitation of Ataxic Cerebral Palsy Children | Zeping Li | Chinese | content not matching |
| 305 | Medical and Educational Integration to Assist Children with Disabilities in Healthy Growth: A Case Study of Xinxin Kindergarten in Xiamen, Fujian | Xiangxiang | Chinese | content not matching |
| 306 | The Effects of Ganglioside Combined with Xieyin Buyang Acupuncture on Neurological Function and Motor Ability in Stroke Patients with Flaccid Paralysis | Yu Wang | Chinese | content not matching |
| 307 | The Effect of Rope Therapy Training on Lower Limb Motor Function in Children with Spastic Cerebral Palsy | Xin Li | Chinese | inconsistent intervention |
| 308 | Clinical Study on the Effect of Whole-Body Vibration Training on Fine Motor Skills in Children with Cerebral Palsy Assisted by Digital OT System | Man Wang | Chinese | inconsistent intervention |
| 309 | The Effect of Hydrotherapy Combined with Games on Intelligence and Motor Function in Children with Developmental Delay | Yueyu Liu | Chinese | inconsistent intervention |
| 310 | Study on the Rehabilitation Effect of Water-Based Sensory Integration Combined with Comprehensive Rehabilitation Treatment in Children with Global Developmental Delay | Funing Gao | Chinese | inconsistent intervention |
| 311 | Research Progress on the Application of Intramuscular Electrotherapy in Pediatric Rehabilitation | Lina Wang | Chinese | content not matching |
| 312 | Systematic Review and Meta-analysis on the Effects of Aquatic Exercise Therapy on Motor Function and Activity in Children and Adolescents with Cerebral Palsy | Yao Cui | Chinese | systematic reviews |
| 313 | Observation on the Efficacy of Biofeedback Combined Therapy in Promoting Lower Limb Motor Function in Children with Spastic Diplegic Cerebral Palsy | Zhongmin Yi | Chinese | content not matching |
| 314 | Observation on the Effect of Tongdu Xinao Yishen Jianpi Massage on Neurological Development in Premature Infants with Brain Injury | Liting Cen | Chinese | content not matching |
| 315 | The Effect of Scalp Acupuncture Combined with Traditional Chinese Medicine Hydrotherapy on Gross Motor Function in Children with Spastic Cerebral Palsy | Dongliang Xiang | Chinese | content not matching |
| 316 | The Effect of Tuina Combined with Spastic Muscle Stimulator on Gross Motor Function in Children with Cerebral Palsy | Jun Xu | Chinese | content not matching |
| 317 | Providing Suitable Education for Every Student | Xianyin Huang | Chinese | content not matching |
| 318 | The Effect of Acupuncture on Motor Function and Visual Evoked Potentials in Children with Spastic Cerebral Palsy | Jian Tang | Chinese | content not matching |
| 319 | Observation on the Efficacy of Lower Limb Traditional Chinese Medicine Bath Combined with Paraffin Therapy in the Treatment of Spastic Cerebral Palsy | Yan'e Shen | Chinese | content not matching |
| 320 | Observation on the Efficacy of Pediatric Rehabilitation Medicinal Bath Formula in the Treatment of Spastic Cerebral Palsy | Fengming Tian | Chinese | content not matching |
| 321 | Analysis and Insights into Rehabilitation Treatment for Children with Cerebral Palsy | Lidai Su | Chinese | content not matching |
| 322 | Current Status and Research Progress in the Rehabilitation Treatment of Pediatric Cerebral Palsy | Yiming Wen | Chinese | systematic reviews |
| 323 | The Effect and Mechanism of Isoflurane Exposure on Long-Term Neurobehavioral Outcomes in Neonatal Rats | Ying Zhao | Chinese | content not matching |
| 324 | The Effect of Brain Awakening Acupuncture Combined with Acupressure on Clinical Efficacy, Intelligence, and Motor Function in Children with Cerebral Palsy | Zhenzhen Liu | Chinese | content not matching |
| 325 | The Clinical Efficacy of Brain Awakening Acupuncture Combined with NGF Acupoint Injection in Children with Brain Injury Syndrome | Zhenhuan Liu | Chinese | content not matching |
| 326 | The Clinical Efficacy of Brain Awakening Acupuncture Combined with NGF Acupoint Injection in Brain Injury Syndrome | Shangsheng Dong | Chinese | content not matching |
| 327 | The Effect of Meridian Acupoint Therapy Combined with Progressive Resistance Training on Lower Limb Motor Function, Muscle Tone, and Gait in Children with Spastic Cerebral Palsy | Guimei Peng | Chinese | content not matching |
| 328 | The Effects of Subanesthetic Dose of Ketamine and Its Combination with Alcohol on Behavior and Neuronal Apoptosis in Pubertal Macaques and Rats | Qing Li | Chinese | animal experiments |
| 329 | The Effect of Community Medical-Educational Combined Service Model on Motor Function in School-Aged Children with Cerebral Palsy | Lianghua Jiang, | Chinese | content not matching |
| 330 | The Effect of Medical Ozone on Hypoxic Brain Injury in Zebra Fish Larvae | Yan Cheng, | Chinese | content not matching |
| 331 | The Effect of Guided Health Education Combined with Sandplay Game Intervention on Intelligence and Motor Ability in Children with Cerebral Palsy | Yuanyuan Tian | Chinese | content not matching |
| 332 | The Effect of Sandplay Game Combined with Guided Education on Motor Development in Children with Cerebral Palsy | Ben Han | Chinese | content not matching |
| 333 | The Effect of Guided Education on Motor Ability in Children with Cerebral Palsy | Hongwei Xu | Chinese | content not matching |
| 334 | The Intervention Effect of Guided Education Combined with Suspension Exercise on Motor Function, Daily Living Ability, and Comprehensive Function in Children with Spastic Cerebral Palsy | Xiaodong Kang | Chinese | content not matching |
| 335 | Application and Effect of Guided Education in the Rehabilitation of Children with Cerebral Palsy – Experience from Implementing the China Disabled Persons' Federation's Emergency Rehabilitation Program for Children with Disabilities in Poverty | Heng Zhang | Chinese | content not matching |
| 336 | Discussion on the Etiology and Prevention of Infantile Cerebral Palsy (with Clinical Analysis of 128 Cases) | Xiaoyu Zhou | Chinese | content not matching |
| 337 | "Infant Swimming" Quality Assessment for Predicting the Later Neurodevelopment of High-Risk Children | Dingmin Wei, | Chinese | content not matching |
| 338 | Protecting the Environment for Our Home | Anonymous | Chinese | content not matching |
| 339 | The Early Intervention Effects of Swimming and Touching on Infant Brain Injury | Wenchun Liu, | Chinese | content not matching |
| 340 | The Effect of Swimming Rehabilitation Training on Certain Biochemical Markers in Children with Cerebral Palsy | Yonghui Fan | Chinese | content not matching |
| 341 | Exploration and Verification of Neural Circuits in Improving Motor Dysfunction in Children with Autism through Motor Intervention | Xuan Xiong | Chinese | content not matching |
| 342 | Discussion on the Efficacy of Comprehensive Rehabilitation Methods in Treating Children with Cerebral Palsy at Different Age Stages | Jiafang Meng | Chinese | content not matching |
| 343 | The Effect of Early Intervention on Intelligence, Motor Ability, and the Occurrence of Cerebral Palsy in Premature Infants | Jianying Li | Chinese | content not matching |
| 344 | Observation on the Effect of Early Rehabilitation Nursing on Motor Ability and Intelligence in Children with Cerebral Palsy | Yali Liu | Chinese | content not matching |
| 345 | The Effect of Early Comprehensive Intervention on Physical and Intellectual Development in Premature Infants | Rongzhi Dong | Chinese | content not matching |
| 346 | The Effect of Acupuncture at the Du Meridian and Jiaji Points on Core Muscle Strength and Motor Function in Children with Involuntary Movement Type Cerebral Palsy | Xuemei Luo | Chinese | content not matching |
| 347 | Observation on the Effect of Acupuncture on Core Muscle Groups Combined with Rehabilitation Training in Treating Cerebral Palsy and Its Effect on Motor and Balance Ability | Qiangli Wang | Chinese | content not matching |
| 348 | Observation on the Efficacy of Acupuncture Combined with Cerebral Ultrasound Therapy in Infant Brain Injury Syndrome | Chiye Zheng | Chinese | content not matching |
| 349 | Observation on the Efficacy of Acupuncture Combined with Traditional Chinese Medicine Bath in the Treatment of Spastic Cerebral Palsy | Yuxin Wang | Chinese | content not matching |
| 350 | The Effect of Acupuncture Combined with Low-Frequency Electrical Stimulation on Lower Limb Muscle Tone in Children with Spastic Cerebral Palsy | Wenjie Fu | Chinese | content not matching |
| 351 | The Effect of Acupuncture Combined with Low-Frequency Electrical Stimulation on Lower Limb Muscle Tone in Children with Spastic Cerebral Palsy | Wenjie Fu | Chinese | content not matching |
| 352 | Exploration and Verification of Neural Circuits in Improving Motor Dysfunction in Children with Autism through Motor Intervention | Xuan Xiong | Chinese | content not matching |
| 353 | Discussion on the Efficacy of Comprehensive Rehabilitation Methods in Treating Children with Cerebral Palsy at Different Age Stages | Jiafang Meng | Chinese | content not matching |
| 354 | The Effect of Early Intervention on Intelligence, Motor Ability, and the Occurrence of Cerebral Palsy in Premature Infants | Jianying Li | Chinese | content not matching |
| 355 | Observation on the Effect of Early Rehabilitation Nursing on Motor Ability and Intelligence in Children with Cerebral Palsy | Yali Liu | Chinese | content not matching |
| 356 | The Effect of Early Comprehensive Intervention on Physical and Intellectual Development in Premature Infants | Rongzhi Dong | Chinese | content not matching |
| 357 | The Effect of Acupuncture at the Du Meridian and Jiaji Points on Core Muscle Strength and Motor Function in Children with Involuntary Movement Type Cerebral Palsy | Xuemei Luo | Chinese | content not matching |
| 358 | Observation on the Effect of Acupuncture on Core Muscle Groups Combined with Rehabilitation Training in Treating Cerebral Palsy and Its Effect on Motor and Balance Ability | Qiangli Wang | Chinese | content not matching |
| 359 | Observation on the Efficacy of Acupuncture Combined with Cerebral Ultrasound Therapy in Infant Brain Injury Syndrome | Chiye Zheng | Chinese | content not matching |
| 360 | Observation on the Efficacy of Acupuncture Combined with Traditional Chinese Medicine Bath in the Treatment of Spastic Cerebral Palsy | Yuxin Wang | Chinese | content not matching |
| 361 | The Effect of Acupuncture Combined with Low-Frequency Electrical Stimulation on Lower Limb Muscle Tone in Children with Spastic Cerebral Palsy | Wenjie Fu | Chinese | content not matching |
| 362 | Clinical Study on Acupuncture Combined with Low-Frequency Electrical Stimulation in the Treatment of Toe Deformity in Children with Spastic Cerebral Palsy | Wenjie Fu | Chinese | content not matching |
| 363 | Clinical and Mechanistic Study on Acupuncture Combined with Rehabilitation Training in the Treatment of Spastic Cerebral Palsy in Children | Yanju Ma | Chinese | content not matching |
| 364 | The Effect of Acupuncture on Monoamine Neurotransmitters and Amino Acids in Brain Tissue of Neonatal Rat CP Model | Yanghaiyan Du | Chinese | animal experiments |
| 365 | The Effect of Combined Traditional Chinese and Western Medicine with Acupuncture and Massage on Symptoms, Hemorheology, and Cerebral Blood Flow Parameters in Patients with Vertebral Artery Type Cervical Spondylosis | Shuguang Yuan | Chinese | content not matching |
| 366 | Clinical Study on the Neurodevelopmental Effects of Zhishiqi in Children with Brain Injury Syndrome | Jieqi Chen | Chinese | content not matching |
| 367 | Observation on the Efficacy of Traditional Chinese Medicine Antispasmodic Bath Combined with Rehabilitation Training in the Treatment of Spastic Cerebral Palsy | Yan Zhang | Chinese | content not matching |
| 368 | Comprehensive Therapy with Traditional Chinese Medicine Oral Administration, Acupuncture, Tuina, Medicinal Bath, and Acupoint Embedding for the Treatment of 20 Cases of Spastic Cerebral Palsy | Xiaohui Yang | Chinese | content not matching |
| 369 | Observation on the Efficacy of Traditional Chinese Medicine Bubble Bath in Relieving Muscle Tone in Spastic Diplegic Cerebral Palsy Children | Dunwu Xiao | Chinese | content not matching |
| 370 | Observation on the Efficacy of Ankle Joint Passive Motion Device Combined with Functional Training in the Treatment of Toe Deformity in Children with Spastic Cerebral Palsy | Yajun Long | Chinese | content not matching |
| 371 | The Effect of Traditional Chinese Medicine Hydrotherapy Combined with Rehabilitation Nursing on Spastic Cerebral Palsy Children | Yanmei He | Chinese | content not matching |
| 372 | Observation on the Effect of Traditional Chinese Medicine Medicinal Bath Combined with Nursing Intervention on Spastic Cerebral Palsy Children | Yingyu Huang | Chinese | content not matching |
| 373 | Clinical Observation on the Treatment of 39 Cases of Spastic Cerebral Palsy Children with Traditional Chinese Medicine Medicinal Bath as the Main Treatment | Yanjuan Hou | Chinese | content not matching |
| 374 | Clinical Observation on the Effect of Traditional Chinese Medicine Bath Combined with Tuina on Ankle Joint Activity in Children with Spastic Cerebral Palsy | Yong Zhao | Chinese | content not matching |
| 375 | Clinical Observation on the Treatment of 25 Cases of Spastic Diplegic Cerebral Palsy Children with Traditional Chinese Medicine Bath Combined with Conventional Rehabilitation Treatment | Yuepeng Lu | Chinese | content not matching |
| 376 | Observation on the Effect of Traditional Chinese Medicine Steam Bath Combined with Functional Training in Reducing Muscle Tone in Children with Spastic Cerebral Palsy | Paoqiu Wang | Chinese | content not matching |
| 377 | The Effect of Traditional Chinese Medicine Tuina and Medicinal Bath on Neuro-Motor Development in Children with Brain Injury Syndrome | Yong Zhang | Chinese | content not matching |
| 378 | Clinical Study on the Comprehensive Traditional Chinese Medicine Treatment for Children with Cerebral Palsy, Liver Qi Stagnation and Spleen Deficiency Syndrome | Maoqing Li | Chinese | content not matching |
| 379 | Research Progress on Motor Function Assessment and Intervention in Children with Autism Spectrum Disorder | Weiwei Chen | Chinese | content not matching |
| 380 | Observation on the Effect of Comprehensive Nursing Intervention on Motor Ability in Children with Cerebral Palsy | Yuqun Wen | Chinese | content not matching |
| 381 | The Application of Comprehensive Rehabilitation Therapy in Children's Welfare Institutions | Weili Dang | Chinese | content not matching |
| 382 | The Effect of Comprehensive Rehabilitation Treatment on the Recovery of Neurological Function and Motor Ability in Children with Cerebral Palsy | Minggao Cheng | Chinese | content not matching |
| 383 | Observation on the Efficacy of Comprehensive Rehabilitation Treatment in High-Risk Children with Cerebral Palsy | Jiujuan Qiu | Chinese | content not matching |
| 384 | The Effect of Crawling Promotion Training Robot on Cognitive and Fine Motor Function in Children with Spastic Diplegic Cerebral Palsy | Yu Hua | Chinese | content not matching |
| 385 | Observation on the Efficacy of Comprehensive Therapy in the Treatment of Scissor Gait in Children with Cerebral Palsy | Qin Qin | Chinese | content not matching |
| 386 | Clinical Study on the Treatment of 50 Cases of Spastic Cerebral Palsy Children with Comprehensive Therapy | Wan Ci Tan | Chinese | content not matching |
| 387 | Clinical Practice Guidelines for Aquatic Exercise Therapy in Children with Cerebral Palsy | Rehabilitation Treatment Group of the Chinese Medical Association Physical Medicine and Rehabilitation Branch | Chinese | systematic reviews |
| 388 | The Effect of Hydrotherapy on Pulmonary Function in Children with Spastic Diplegic Cerebral Palsy | Wenjie Hao | Chinese | content not matching |
| 389 | The Effect of Hydrotherapy on Respiratory Function in Children with Cerebral Palsy (Review) | Wen Zhi Du | Chinese | systematic reviews |

| Reports not retrieved(n =17) | | | | |
| --- | --- | --- | --- | --- |
| No. | Study Title (Translated) | First Author. | Language | Exclusion Reason |
| 1 | Aquatic-based Explosive Strength Training in Children With Cerebral Palsy | Cairo University | English | reports not retrieved |
| 2 | Benefits aquatic physical therapy in the trunk control in children with cerebral palsy | Mirna Sayuri Kanashiro | English | reports not retrieved |
| 3 | Does the Acquisition of Water Orientation Skills Influence the Abilities of Moving on Land and Improve Balance on Land? | DA Maniu | English | reports not retrieved |
| 4 | Effectiveness of occupational therapy focussing on upper extremity training in a temperature controlled pool versus land | Sharan D | English | reports not retrieved |
| 5 | Effects of aquatic exercises in trunk control and their interference in gait of subjects with cerebral palsy | Luize Bueno de Araujo | English | reports not retrieved |
| 6 | Effects of Aquatic Therapy as an Addition to Conventional Rehabilitation Therapy on Motor Function among Preschool Children with Spastic Cerebral Palsy | ChiCtr | English | reports not retrieved |
| 7 | Effects Of Aquatic Therapy On Motor Function Among Preschool Children With Spastic Cerebral Palsy: 512 | H Zhang | English | reports not retrieved |
| 8 | Effects of virtual rehabilitation, bobath concept, and aquatic therapy in children with cerebral palsy | Martins, L. G. | English | reports not retrieved |
| 9 | The Impact of Aquatic Therapy on Walking, Balance Functions, and Quality of Life in Children With Cerebral Palsy | Ankara City Hospital Bilkent | English | reports not retrieved |
| 10 | Impact of underwater treadmill training on walking performance in youth with cerebral palsy: C5 | D Morgan | English | reports not retrieved |
| 11 | Posture and balance in children with cerebral palsy under different therapeutic approaches | F Maciel | English | reports not retrieved |
| 12 | Profusely increased cortical grey, subcortical grey and white matter structural neuroplasticity with enduring real-world motor improvement following CI therapy for MS: Results from a randomised controlled trial | Mark, V. W. | English | reports not retrieved |
| 13 | Training of arms on body alignment individuals with cerebral palsy: a pilot study | Edlaine Ribeiro Oliveira | English | reports not retrieved |
| 14 | Trunk Control in an Aquatic Environment for Children With Cerebral Palsy: randomized Clinical Pilot Trial...5th International Conference For Evidence Based Aquatic Therapy (ICEBAT), Apr 14-16, 2018, Las Vegas, Nevada | Kakihata AM | English | reports not retrieved |
| 15 | The Effect of Hydrotherapy on Gross Motor Development in Infants with Motor Developmental Delay Aged 0-1 | Jianqun Lei | Chinese | reports not retrieved |
| 16 | Clinical Observation on the Treatment of Spastic Cerebral Palsy with Hydrotherapy Combined with Bobath Method | Yuting Zou | Chinese | reports not retrieved |
| 17 | Effects of Pediatric Aquatic Therapy in Children With Spastic Cerebral Palsy | Chang Gung Memorial Hospital | English | reports not retrieved |

| **Reports excluded(NO access to full therapy,Study design)(n =26)** | | | | |
| --- | --- | --- | --- | --- |
| **No.** | **Study Title (Translated)** | **First Author.** | **Language** | **Exclusion Reason** |
| 1 | The Effect of Hydrotherapy on the Rehabilitation of Motor Function in Children with Delayed Cerebral Palsy | Yanxia Gu | Chinese | no access to full therapy |
| 2 | A Study on the Effect of Hydrotherapy on Children with Developmental Delay and Central Coordination Disorder | Weiwei Cao | Chinese | no access to full therapy |
| 3 | Observation on the Effect of Hydrotherapy in Children with Spastic Cerebral Palsy | Meiju Song | Chinese | no access to full therapy |
| 4 | Observation on the Rehabilitation Effect of Hydrotherapy in Children with Spastic Cerebral Palsy | Xiumin Yuan | Chinese | no access to full therapy |
| 5 | Effect Analysis of Hydrotherapy Intervention on Gross Motor Function in Children with Central Coordination Disorder | Huiying Du | Chinese | no access to full therapy |
| 6 | Observation on the Effect of Hydrotherapy Rehabilitation Training in Children with Cerebral Palsy | Huanping Shen | Chinese | no access to full therapy |
| 7 | The Treatment of 130 Cases of Children with Cerebral Palsy by Hydrotherapy Combined with Comprehensive Therapy | Hongyan Wu | Chinese | no study design |
| 8 | Progress in the Application of Aquatic Exercise Therapy in Children with Cerebral Palsy | Jing Zhang, | Chinese | no access to full therapy |
| 9 | The Effect of Using Guided Education During Aquatic Exercise on Cerebral Palsy Treatment | Jigge Dong | Chinese | no study design |
| 10 | The Rehabilitation Effect of Swimming on School-Aged Children with Cerebral Palsy | Chuyang Li | Chinese | no access to full therapy |
| 11 | A Study on the Effect of Hydrotherapy in Reducing Muscle Tone in Children with Spastic Cerebral Palsy | Suxia Zhang | Chinese | no access to full therapy |
| 12 | Analysis of 89 Cases of Hydrotherapy and Acupuncture Comprehensive Therapy to Improve Muscle Tone in Children with Cerebral Palsy | Xiaoqin Zhang | Chinese | no access to full therapy |
| 13 | The Effect of "Bubble Water Bath" Treatment for Children with Spastic Cerebral Palsy | Hongying Liu | Chinese | no access to full therapy |
| 14 | Observation on the Effect of Hydrotherapy in Reducing Muscle Tone in Children with Spastic Cerebral Palsy | Miao Wang | Chinese | no access to full therapy |
| 15 | The Effect of Halliwick Technique on Balance and Gross Motor Function in School-Aged Children with Cerebral Palsy | Xiaohui Hou | Chinese | no study design |
| 16 | Combined Effect of Hydrotherapy and Transcranial Direct-Current Stimulation on Children with Cerebral Palsy: A Protocol for a Randomized Controlled Trial | X.L. Chen | English | no study design |
| 17 | Group Swimming and Aquatic Exercise Programme for Children with Autism Spectrum Disorders: A Pilot Study | M.A. Fragala-Pinkham | English | no study design |
| 18 | Pediatric Aquatic Therapy on Motor Function and Enjoyment in Children Diagnosed with Cerebral Palsy of Various Motor Severities | C.J. Lai | English | no study design |
| 19 | Neuromechanism, Recovery Effect, and Case Study of Swimming Training Intervention in Children with Cerebral Palsy: A Case Report | J. Zeng | English | no study design |
| 20 | Rehabilitation of Spastic Tetraplegia in Pediatrics: Advantages of Hydro-Kinesitherapy | F. Dervishaliaj | English | no study design |
| 21 | Group Aquatic Training Improves Gait Efficiency in Adolescents with Cerebral Palsy | L. Ballaz, S. | English | no study design |
| 22 | The Effect of Eight Weeks of Aquatic Exercises on Muscle Strength in Children with Cerebral Palsy: A Case Study | M. Esmailiyan | English | no study design |
| 23 | Effects of Aquatic Aerobic Exercise for a Child with Cerebral Palsy: Single-Subject Design | R. Retarekar | English | no study design |
| 24 | Evaluating the Effects of Performance-Focused Swimming Training on People with Cerebral Palsy Who Have High Support Needs – A Study Protocol Using Single-Case Experimental Design | I.M. Dutia | English | no study design |
| 25 | Flexibility Evaluation by the Method of Wells' Flexometer in Children with Cerebral Palsy Submitted to Hydrotherapy Treatment: Study of the Cases | [AP Espindula](https://scholar.google.com.hk/citations?user=hctWhPgAAAAJ&hl=zh-CN&oi=sra) | English | no study design |
| 26 | Influence of Aquatic Therapy in Children and Youth with Cerebral Palsy: A Qualitative Case Study in a Special Education School | E. Muñoz-Blanco | English | no study design |

| **Reports of included studies(n =14)**  **Records identified from:**  **RCT experiments obtained from other papers (n = 2)** | | | | |
| --- | --- | --- | --- | --- |
| **No.** | **Study Title (Translated)** | **First Author.** | **Language** | **Reports of included studies** |
| 1 | The effect of aquatic exercise on spasticity, quality of life, and motor function in cerebral palsy | Adar, S. | English | reports of included studies |
| 2 | Aquatic Exercise Intervention Is Effective for Spasticity Inhibition in Children with Cerebral Palsy: A Clinical Controlled Study | Akinola, B. I. | English | reports of included studies |
| 3 | Comparing the effects of aquatic and land-based exercises on balance and walking in spastic diplegic cerebral palsy children | Badawy | English | reports of included studies |
| 4 | Effects of an aquatic program on gross motor function of children with spastic cerebral palsy | Chrysagis | English | reports of included studies |
| 5 | Benefits and enjoyment of a swimming intervention for Youth with Cerebral Palsy: an RCT Study | Declerck, M. | English | reports of included studies |
| 6 | The effect of aquatic intervention on the gross motor function and aquatic skills in children with cerebral palsy | Dimitrijević, L | English | reports of included studies |
| 7 | The effects of Halliwick aquatic exercises on gross motor function of children aged from 3 to 5 years with spastic cerebral palsy | Hamed, S. A | English | reports of included studies |
| 8 | Comparison between the effect of aquatic exercise program and land exercise program in spastic cerebral palsy on motor function and balance | Kang, S. | English | RCT experiments obtained from other papers |
| 9 | The effectiveness of a specialized hydrotherapy program based on Halliwick concept in the transition from supine to sitting of children with cerebral palsy: a randomised control trial | Konstantinos Chandolias | English | reports of included studies |
| 10 | Analysis of the effect of functional hydrotherapy on muscle strength and motor function in children with spastic cerebral palsy | Wenwen Luo | Chinese | RCT experiments obtained from other papers |
| 11 | Impact of aquatic exercise program on muscle tone in spastic hemiplegic children with cerebral palsy | Olama, K. A. | Chinese | reports of included studies |
| 12 | Effects of hydrotherapy on gross motor function and lower limb muscle strength and tone in children with spastic diplegic cerebral palsy | Song Fanxu | Chinese | reports of included studies |
| 13 | Effects of aerobic hydrotherapy on movement, balance function, and muscle tension of spastic cerebral palsy children | Zhao Yonghong | Chinese | reports of included studies |
| 14 | Effect of Halliwick Therapy on Lower Limb Motor Skills in Children with Spastic Cerebral Palsy | Zhang Juan | Chinese | reports of included studies |
| 15 | Effect of hydrotherapy intervention on lower limb motor skills of children with spastic cerebral palsy | Zhu Qing | Chinese | reports of included studies |
| 16 | Effect of aquatic motor therapy combined with routine rehabilitation therapy on motor function among children with spastic cerebral palsy | Zhong Chen | Chinese | reports of included studies |
