# Supplementary material for: The effects of hydrotherapy on athletic ability in children with cerebral palsy: A systematic review and meta-analysis
Source: PLoS One. 2025 Jun 10;20(6):e0325517. doi: 10.1371/journal.pone.0325517 (PMC12151372; doi:10.1371/journal.pone.0325517)
Supplement: S2 Data — (DOCX) [file pone.0325517.s005.docx]

**S5 Data availability statement**

| **Raw data on the effect of hydrotherapy on gross motor functions** | | | | |
| --- | --- | --- | --- | --- |
| Study Author | Hydrotherapy Group Mean | Hydrotherapy Group SD | Conventional Training Group Mean | Conventional Training Group SD |
| Adar2017 | 202.1 | 63.3 | 208 | 38.75 |
| Chrysagis2009 | 69.155 | 5.1025 | 64.035 | 0.131225 |
| Dimitrijevic2012 | 73.04 | 27.44 | 66.56 | 29.84 |
| Hamed2023 | 0.29 | 0.22 | 0.22 | 0.11 |
| Chandolias.K 2022 | 75.757 | 12.3766 | 65.308 | 20.6663 |
| Luo Wenwen2018 | 47.2 | 11.4 | 39.5 | 12.7 |
| Song Fanxu2015 | 44.02 | 11.67 | 39.85 | 10.93 |
| Sung-Hoon Kang2012 | 62.9 | 19.76 | 64.24 | 17.16 |
| Zhang Juan2021 | 226.81 | 27.98 | 221.87 | 34.76 |
| Zhao Yonghong2021 | 79.27 | 6.75 | 68.64 | 7.44 |
| Zhong Chen2023 | 30.735 | 4.06 | 32.31 | 3.8 |
| Zhu Qing2022 | 245.09 | 31.54 | 231.59 | 34.4 |

| **Raw data on the effect of hydrotherapy on gross motor functions children aged 6 years and children** | | | | |
| --- | --- | --- | --- | --- |
| Study Author | Hydrotherapy Group Mean | Hydrotherapy Group SD | Conventional Training Group Mean | Conventional Training Group SD |
| Adar2017 | 202.1 | 63.3 | 208 | 38.75 |
| Chrysagis2009 | 69.155 | 5.1025 | 64.035 | 0.131225 |
| Dinitrijevic2012 | 73.04 | 27.44 | 66.56 | 29.84 |
| Chandolias.K 2022 | 75.757 | 12.3766 | 65.308 | 20.6663 |
| Luo Wenwen2018 | 47.2 | 11.4 | 39.5 | 12.7 |
| Sung-Hoon Kang2012 | 62.9 | 19.76 | 64.24 | 17.16 |

| **Raw data on the effect of hydrotherapy on gross motor functions in children aged over 6 years** | | | | |
| --- | --- | --- | --- | --- |
| Study Author | Hydrotherapy Group Mean | Hydrotherapy Group SD | Conventional Training Group Mean | Conventional Training Group SD |
| Hamed2023 | 0.29 | 0.22 | 0.22 | 0.11 |
| Song Fanxu2015 | 44.02 | 11.67 | 39.85 | 10.93 |
| Zhang Juan2021 | 226.81 | 27.98 | 221.87 | 34.76 |
| Zhao Yonghong2021 | 79.27 | 6.75 | 68.64 | 7.44 |
| Zhong Chen2023 | 30.735 | 4.06 | 32.31 | 3.8 |
| Zhu Qing2022 | 245.09 | 31.54 | 231.59 | 34.4 |

| **Raw data on the effect of hydrotherapy on gross motor functions with a treatment duration of no more than 10 weeks** | | | | |
| --- | --- | --- | --- | --- |
| Study Author | Hydrotherapy Group Mean | Hydrotherapy Group SD | Conventional Training Group Mean | Conventional Training Group SD |
| Adar2017 | 202.1 | 63.3 | 208 | 38.75 |
| Chrysagis2009 | 69.155 | 5.1025 | 64.035 | 0.131225 |
| Dinitrijevic2012 | 73.04 | 27.44 | 66.56 | 29.84 |
| Sung-Hoon Kang2012 | 62.9 | 19.76 | 64.24 | 17.16 |

| **Raw data on the effect of hydrotherapy on gross motor functions with a treatment duration of more than 10 weeks** | | | | |
| --- | --- | --- | --- | --- |
| Study Author | Hydrotherapy Group Mean | Hydrotherapy Group SD | Conventional Training Group Mean | Conventional Training Group SD |
| Chandolias.K 2022 | 75.757 | 12.3766 | 65.308 | 20.6663 |
| Luo Wenwen2018 | 47.2 | 11.4 | 39.5 | 12.7 |
| Hamed2023 | 0.29 | 0.22 | 0.22 | 0.11 |
| Song Fanxu2015 | 44.02 | 11.67 | 39.85 | 10.93 |
| Zhang Yonghong | 79.27 | 6.75 | 68.64 | 7.44 |
| Zhang Juan2021 | 226.81 | 27.98 | 221.87 | 34.76 |
| Zhong Chen2023 | 30.735 | 4.06 | 32.31 | 3.8 |
| Zhu Qing2022 | 245.09 | 31.54 | 231.59 | 34.4 |

| **Raw data on the effect of hydrotherapy on fine motor functions** | | | | |
| --- | --- | --- | --- | --- |
| Study Author | Hydrotherapy Group Mean | Hydrotherapy Group SD | Conventional Training Group Mean | Conventional Training Group SD |
| Luo Wenwen2015 | 30.6 | 6.2 | 26.8 | 7 |
| Zhang Juan2021 | 72.76 | 10.87 | 62.25 | 9.76 |

| **Raw data on the effect of hydrotherapy on balance** | | | | |
| --- | --- | --- | --- | --- |
| Study Author | Hydrotherapy Group Mean | Hydrotherapy Group SD | Conventional Training Group Mean | Conventional Training Group SD |
| Badawy2015 | -1.7 | 0.29 | -2.31 | 0.28 |
| Sung-Hoon Kang2012 | 4 | 14.76 | 3.21 | 11.32 |
| Zhang Jun2021 | 42.13 | 24.21 | 32.12 | 14.73 |
| Zhao Yonghong2021 | 78.17 | 7.46 | 76.9 | 6.99 |

| **Raw data on the effect of hydrotherapy muscle tone** | | | | |
| --- | --- | --- | --- | --- |
| Study Author | Hydrotherapy Group Mean | Hydrotherapy Group SD | Conventional Training Group Mean | Conventional Training Group SD |
| Adar2017 | 0 | 0.25 | 0 | 1 |
| Song Fanxu2015 | 2.35 | 1.11 | 3.26 | 0.8 |
| Sung-Hoon Kang2012 | 0.4 | 0.79 | 0.39 | 0.83 |
| Zhong Chen2023 | 1.42 | 0.51 | 1.9 | 0.64 |
